# Unleashing innovation: 3D-printed biomaterials in bone tissue engineering for repairing femur and tibial defects in animal models – a systematic review and meta-analysis

**DOI:** 10.3389/fbioe.2024.1385365

**Published:** 2024-09-23

**Authors:** Nitin Sagar, Bandana Chakravarti, Shailendra S. Maurya, Anshul Nigam, Pushkar Malakar, Rajesh Kashyap

**Affiliations:** ^1^ Stem Cell Research Centre, Department of Hematology, Sanjay Gandhi Postgraduate Institute of Medical Sciences, Lucknow, India; ^2^ Center for Advanced Research (Stem Cell/Cell Culture Lab), King George’s Medical University, Lucknow, India; ^3^ Department of Biotechnology, Kanpur Institute of Technology, Kanpur, India; ^4^ Department of Biomedical Science and Technology, School of Biological Sciences, Ramakrishna Mission Vivekananda Educational Research Institute (RKMVERI), Kolkata, India; ^5^ Department of Hematology, Sanjay Gandhi Postgraduate Institute of Medical Sciences, Lucknow, India

**Keywords:** bone repair, 3D printing, biomaterials, meta-analysis, bone defects

## Abstract

**Introduction:**

3D-printed scaffolds have emerged as an alternative for addressing the current limitations encountered in bone reconstruction. This study aimed to systematically review the feasibility of using 3D bio-printed scaffolds as a material for bone grafting in animal models, focusing on femoral and tibial defects. The primary objective of this study was to evaluate the efficacy, safety, and overall impact of these scaffolds on bone regeneration.

**Methods:**

Electronic databases were searched using specific search terms from January 2013 to October 2023, and 37 relevant studies were finally included and reviewed. We documented the type of scaffold generated using the 3D printed techniques, detailing its characterization and rheological properties including porosity, compressive strength, shrinkage, elastic modulus, and other relevant factors. Before incorporating them into the meta-analysis, an additional inclusion criterion was applied where the regenerated bone area (BA), bone volume (BV), bone volume per total volume (BV/TV), trabecular thickness (Tb. Th.), trabecular number (Tb. N.), and trabecular separation (Tb. S.) were collected and analyzed statistically.

**Results:**

3D bio-printed ceramic-based composite scaffolds exhibited the highest capacity for bone tissue regeneration (BTR) regarding BV/TV of femoral and tibial defects of animal models. The ideal structure of the printed scaffolds displayed optimal results with a total porosity >50% with a pore size ranging between 300- and 400 µM. Moreover, integrating additional features and engineered macro-channels within these scaffolds notably enhanced BTR capacity, especially observed at extended time points.

**Discussion:**

In conclusion, 3D-printed composite scaffolds have shown promise as an alternative for addressing bone defects.

## 1 Introduction

Bone tissue constitutes approximately 15% of total body weight and consists of two distinct layers: the outer layer, known as cortical bone, boasts high mechanical strength, while the inner layer, spongy bone, exhibits significant porosity, with a coefficient ranging from 80% to 90% (Dec et al., 2022). The hardness of bone is attributed to its extracellular collagen matrix, which is infused with inorganic calcium phosphate molecules, primarily hydroxyapatite (Ca10(PO4)6(OH)2) (Jeong et al., 2019). Bone tissue is metabolically active and constantly undergoes resorption and remodeling process. Bone tissue has a natural ability to regenerate itself that is adequate for repairing minor bone defects like cracks and certain types of fractures (Dimitriou et al., 2011). However, larger bone defects (>2 cm or 50% loss of bone circumference) are caused by various factors like trauma, tumor removal, or age-related conditions that can lead to issues like incomplete fusion, abnormal fusion, or pathological fractures (Dumont and Exner, 2009). These larger bone defects cannot heal independently but require clinical interventions for a complete healing process (Dimitriou et al., 2011; Dumont and Exner, 2009). The present gold-standard treatments for addressing substantial bone defects involve bone fixation through biologically inert metallic devices and employing bone autografts and allografts. Nevertheless, these approaches are fraught with inherent risks encompassing potential disease transmission and the intricate healing processes that affect both the recipient patients and the donor sites. To address these challenges, bone tissue engineering, an interdisciplinary field, integrates knowledge from cell biology, engineered materials, and biochemical factors (Xue et al., 2022). Utilizing suitable biomaterials, scaffolds or templates is essential to sustain injured tissues or expedite their regeneration. Various fabrication methods have been employed to create these templates, such as salt-leaching, solvent-casting, phase separation, gas foaming, and freeze-drying.

Recently, three-dimensional (3D) printing, referred to as additive manufacturing, is a tool that entails the building of 3D solid objects from a digital file. Conventional 3D printing cannot integrate living components, which limits its relevance in biological contexts (Tripathi et al., 2020). However, this technological advancement has facilitated the emergence of 3D bio-printing, a ground-breaking field in which biological materials are precisely deposited layer by layer to fabricate complex biological structures with potential applications in tissue engineering, synthetic biology, micro/nanofabrication, and regenerative medicine (Ramadan and Zourob, 2020). 3D printers can be categorized into three main types of printing systems: inkjet printers, laser-assisted printers, and micro extrusion printers (Ventola, 2014; Agarwal et al., 2023). While they all involve coordinated spatial motion, they vary in their bioink dispensing methods. The choice of the appropriate printing system should consider factors such as surface resolution, the selection of biological materials, and considerations related to cell viability. 3D bioprinting is regarded as a promising method for the fabrication of biomaterials, scaffolds, or personalized templates and involves the simultaneous printing of biomaterials and cells. Importantly, it enables the creation of intricately porous structures with excellent interconnectedness, allowing for the swift and consistent manufacture of templates tailored to specific or intricate anatomical shapes. This method offers a potential solution for the organ transplant shortage, allowing the fabrication of functional organs like hearts, livers, kidneys, lungs, cartilage, bone, and skin. These 3D bio-printed tissues and organs offer distinct advantages over traditional implants and transplants due to their closer resemblance to biological systems (Panda et al., 2022). Importantly, they significantly reduce the risk of immune system rejection, thereby enhancing the success rate of organ transplantation.

In bone tissue engineering, additive manufacturing starts with the creation of a 3D model of the desired scaffold using computer-aided design (CAD) software. The digital design can be customized to match the patient’s specific anatomical requirements, ensuring the exact geometry of the bone defect or structure to be repaired. The biomaterials utilized in 3D printing for *in vivo* applications in bone tissue engineering must be printable, biocompatible, biodegradable, non-toxic, and capable of providing adequate mechanical strength. Various prototypes of biodegradable scaffolds have been developed using printed polymers, ceramics, and composite for bone tissue regeneration (BTR) and implanted into the femur and tibia of animal models (McGovern et al., 2018; Alonso-Fernandez et al., 2023). The utilization of degradable biomaterials in medicine commenced in 1969 with polymeric biomaterials. The US Food and Drug Administration (FDA) approved various biomaterials for bone tissue engineering such as polyglycolide (PGA), polylactide (PLA), and their co-polymers (PLGA) in varying ratios (Ulery et al., 2011). Some of these polymeric biomaterials have been combined with osteogenic cells or functionalized with bioactive molecules or growth factors to enhance bone regeneration in the femoral and tibial defects of animal models. Another generation of biomaterials, bioactive glasses (BG) and calcium phosphate (CaP)-based bioceramics are widely used in bone tissue engineering due to their excellent bioactivity, osteoconductive and compositional similarities to the bone (Hou et al., 2022). CaP-based bioceramics include β-tricalcium phosphate (β-TCP) and hydroxyapatite (HA) are well-known bone grafting materials due to their resemblance to the bone mineral phase. β-TCP and HA are used for the repair of bone defects in animal models when used alone or in the form of composites with other polymers due to their bioresorbable properties.

Consequently, 3D bioprinting has emerged as a potent tool for manufacturing scaffolds in the field of bone tissue engineering. We conducted this systematic review and meta-analysis to determine the current state of the field of 3D bio-printed scaffolds or templates in bone tissue regeneration of the femur and tibial defects of animal models. We also discussed the challenges associated with transitioning 3D bioprinting technology from the laboratory to the clinical setting.

## 2 Materials and methods

We conducted this study according to the protocols adopted in published systematic reviews and meta-analysis (Rajput et al., 2023).

### 2.1 Systematic search strategy

**FIGURE 1:**
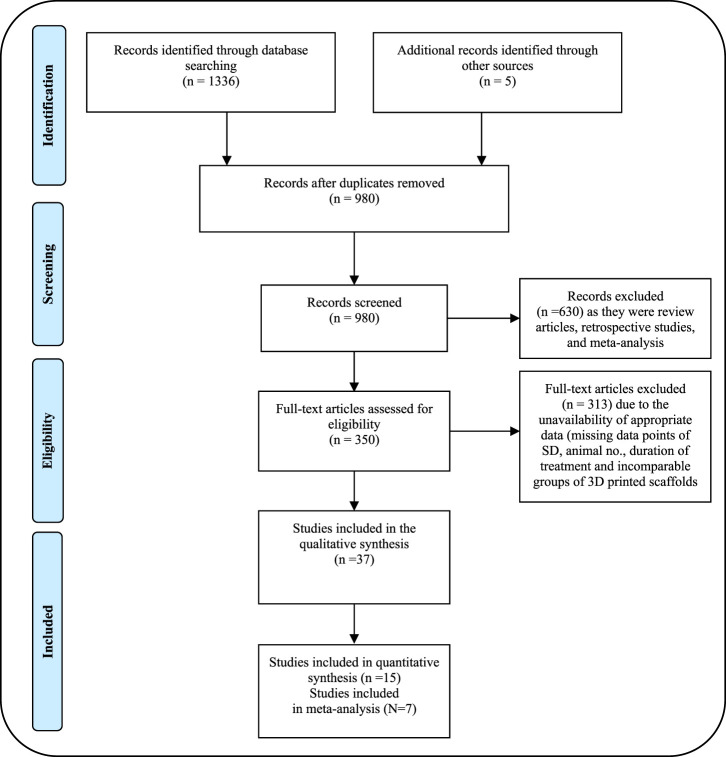
PRISMA flow diagram showing the process of identifying studies chosen for quantitative meta-analysis.

A systematic search for relevant articles was performed by the recommendation of the Preferred Reporting Items for Systematic Reviews and Meta-Analyses (PRISMA) guidelines (Figure 1) to evaluate the impact of 3D printed scaffolds in bone tissue engineering of femoral and tibial defects of animal models. The preclinical studies were identified through a systematic search across electronic databases including PubMed, Web of Science, and Google Scholar, published from January 2013 to October 2023. The terms used for the search included “3D bioprinting, bone, animal studies,” “3D bioprinting, bone, *in vivo* studies,” “3D bioprinting, bioink, animal models,” “3D bioprinting, bioink, bone tissue engineering,” “3D bioprinting, bone tissue engineering, animal studies,” “3D bioprinting, biomaterials, femur defects, *in vivo*,” “3D bioprinting, biomaterials, tibia defects, *in vivo*,” and “3D bioprinting, bone, clinical studies.” The results of the literature review and study screening are displayed in the PRISMA flow diagram in Figure 1.

### 2.2 Study inclusion and exclusion criteria

The inclusion criteria were as follows:

Articles published from January 2013 to October 2023, on the utilization of 3D bioprinting-based scaffolds in bone tissue engineering of femoral and tibial defects in animal models (rat, dog, rabbit, sheep, and pig) were included. The database collection strategy was kept broad to avoid the exclusion of any relevant papers. The literature search results of quantitative data are outlined in the PRISMA flow chart (Figure 1). Inclusion criteria were discussed to assess the relevance of the included *in vivo* studies in the meta-analysis study and to minimize data heterogenicity. Data extraction encompassed a range of information, including but not limited to descriptions of the cells utilized, and materials employed for constructing the structures. Evaluation methods for tissue characterization such as µCT scan, histological, and histomorphometric analysis were assessed for proof-of-concept. Data from animal models was also included. The material characterization for each scaffold, rheological properties (porosity %, compressive modulus, and other relevant parameters) as well as qualitative details of animal models with the femur and tibial defects were extracted from each study included in Supplementary Table S1.

The exclusion criteria were as follows:

1) Articles published before 2013, 2) review articles, 3) short communications, 4) duplication information, 4) case reports, 5) articles written in non-English languages, 6) articles that do not meet the definition of 3D bioprinting or were published in a journal with impact factor (IF, Clarivate) < 1 were excluded. In addition, all *in vivo* studies on 3D-printed templates for craniofacial, mandibular reconstructions, calvarial, and tumor-associated bone defects were also excluded.

### 2.3 Study selection

In this systematic review, two authors (NS and AN) independently searched PubMed, Web of Science, and Google Scholar for all studies. The bibliographies of relevant articles were studied to identify further relevant publications. Titles were initially screened to exclude duplicates and further screened using the abstracts against inclusion and exclusion criteria. Finally, a full-text review of the remainder was performed to assess eligibility.

### 2.4 Data extraction and main outcomes

Two investigators (NS and AN) independently conducted literature screening and disagreements were resolved by discussion with other authors. The data were extracted in numeric form from bar plots of each article using the WebPlotDigitilizer program and from the tables according to PRISMA guidelines. The data were presented in a Microsoft Excel spreadsheet (Windows 10 edition; Microsoft Corporation, Lisbon, Portugal) to record the author name with publication year, types of scaffolds, material characterization, cell type, *in vitro* assay (cell viability), rheological properties, animal models for bone defects such as femur or tibia and defect size, sample size and duration of treatment. Data extracted from all articles included in the meta-analysis is shown in Table 2. The quantitative assessment of the regeneration of femur and tibial defects for each scaffold was calculated and collected for animal models including rabbits, pigs, sheep, dogs, and rats (Table 2). The primary outcome of this study includes the evaluation of immediate and long-term bone repair by analyzing bone volume to tissue volume ratio (BV/TV), bone mineral density (BMD), bone volume (BV), new bone area (NBA), trabecular thickness (Tb. Th.), trabecular number (Tb. N.), and trabecular separation (Tb. S.) using histological or radiographic methods (Table 2).

### 2.5 Quantitative data analysis

Cochran’s Q test and heterogeneity index (I^2^) were used for assessing heterogeneity across studies (Rajput et al., 2023). Due to the low stringency of the heterogeneity test, *p* values <0.10 were considered statistically significant. I^2^ heterogeneity scale of low heterogeneity (<25%), moderate heterogeneity (50%), and high heterogeneity (>75%) was used for quantitative assessment. The pooled effect size was computed using the fixed effect and random effects models and one of the models was adopted depending upon the quantum of heterogeneity. Pooled data analysis was performed using the Comprehensive Meta-Analysis Software (CMA). A sub-group analysis was performed, stratifying the data based on the type of animals and bone defects (whether femoral or tibial defects).

### 2.6 Sensitivity analysis

Sensitivity analysis was performed based on the exclusion of one study at a time. For this, the pooled effect size was computed upon the exclusion of studies one at a time to estimate the sensitive nature of a particular study.

### 2.7 Publication bias analysis

We undertook publication bias analysis qualitatively based on asymmetry in the funnel plot and quantitatively based on Egger’s intercept test and Begg and Mazumdar rank correlation test respectively. In the presence of publication bias, the adjusted values from Duval and Tweedie’s trim and fill method were used to conclude.

## 3 Results

### 3.1 Search results and study selection for different parameters

A total of 1336 potential articles were identified from the literature search. As shown in Figure 1, after reviewing abstracts and titles, 350 potentially eligible studies of animal models for bone defects were assessed carefully by full-text review. 313 studies were excluded due to the unavailability of full text and useful information on 3D bioprinting in bone tissue engineering. Finally, 37 studies of 3D bio-printed scaffolds were considered to meet the inclusion criteria and included in the systematic review and meta-analysis to investigate the advantage of scaffolds in bone tissue regeneration, specifically focusing on animal models with femoral and tibial defects. Among the selected studies, 25 were evaluated for femoral bone defects, while 12 were considered for tibial bone defects in animal models. We summarized the qualitative data from selected studies, providing the details of scaffolds in Supplementary Table S1 and detailed information of animal models with bone defects in Table 1. Notably, we extracted the quantitative data encompassing BV/TV, NBA, Tb. Th., Tb. N., Tb. S. and BV from only 15 out of the 35 studies involving animal models included in the systematic review. Details for the inclusion of studies in the meta-analysis, aligning with the inclusion criteria checklist mentioned earlier, have been incorporated into Table 2. Of these selected studies, 7 studies were conducted to analyze BV/TV specifically for femoral defects (Liu et al., 2016; Lin et al., 2016; Feng et al., 2021; Fairag et al., 2021; Chai et al., 2022; Lei et al., 2023; Ho et al., 2022), and 3 studies focused on tibial defects in animal models (Zhang et al., 2017; Li et al., 2021; Teotia et al., 2020). Among these, 2 studies were excluded due to incomparable control and experimental groups (Chai et al., 2022; Li et al., 2021). Furthermore, for the analysis of NBA, 3 studies about the femur and 2 studies related to the tibia in animal models were selected (Feng et al., 2021; Lei et al., 2023; Tarafder et al., 2015). Notably, 5 studies were undertaken to analyze trabecular thickness Tb. Th., Tb. N., Tb. S. and BV (Fairag et al., 2021; Chai et al., 2022; Ho et al., 2022; Zhang et al., 2017; Li et al., 2021). Two studies were excluded from this analysis due to the incomparable groups of control vs. treatment (Chai et al., 2022; Li et al., 2021). The sample size in these studies ranged from 3 to 15 per group, and treatment duration ranged from 4 weeks to 16 weeks. Subsequently, a meta-analysis was conducted to assess the impact of composite scaffolds on bone tissue regeneration, including parameters such as BV/TV, NBA, Tb. Th. and BV. We used the random-effects model for making inferences due to significant heterogeneity across the studies unless stated otherwise. The pooled and subgroup analyses of all parameters including BV/TV, NBA, Tb. Th and BV have been summarized in Table 3. Most of the studies employed a scaffold-only approach as their control, while their treatment groups utilized 3D-printed composite scaffolds. However, it’s important to note that some studies used a sham as a control which was not included in our analysis.

**TABLE 1:** Methodological characteristics of the studies included in the systematic reviews and meta-analysis for femoral and tibial defects *in vivo* (n = 37 studies).

S.No.	Scaffolds	Animal models	Defect size	Animals detail	Treatments (weeks)	Evaluation method	Outcomes	Included in quantitative data	References
1	SiO2 and ZnO dopants in 3D printed β-TCP scaffolds	Murine Femoral defects	bicortical defect 2.5 mm diameter	SD rats (N = 24, 280–300 g)	4 weeks 6 weeks 8 weeks 12 weeks 16 weeks	Histology Histomorphometry	Enhances osteoinductive properties of CaPs	Yes	Fielding and Bose (2013)
2	Microwave-sintered 3D printed TCP scaffolds	Femoral defects	2–3 mm diameter	SD rats (280–320 g)	2 weeks 4 weeks	Histomorphology	Enhances bone tissue repair and regeneration	No	Tarafder et al. (2013)
3	Bioceramic customized cage	CRCL deficient stifle joints	-	Beagle Dog	1 day 4 weeks 6 weeks 16 weeks	Radiographs	A promising method for the future fabrication of patient-specific bone implants	No	Castilho et al. (2014)
4	SrO- and MgO- in 3D printed Microwave-sintered TCP scaffolds	Rabbit femoral condyle defect model	5 mm diameter and 8 mm depth	NZW rabbits (N = 6, 3.5 kg)	8 weeks 12 weeks	Histomorphology Histomorphometry	Potential for early wound healing through accelerated osteogenesis and vasculogenesis	Yes	Tarafder et al. (2015)
5	Akermanite (Ca2MgSi2O7) scaffold	rabbit femur defect model	critical size circular defect: (Φ-6x6mm)	NZW male rabbit (N = 20, 2.5–3.0 kg)	6 weeks 12 weeks	µCT scan Histology	Enhances tissue regeneration and repair of load-bearing bone defects	Yes	Liu et al. (2016)
6	CHA composite material (ratio-1:2)	rabbit femoral condyle defect model	Φ = 5 mm, L > 10 mm)	NZW rabbits (male, 12 weeks old, 3.25 ± 0.25 kg)	2 weeks 4 weeks 8 weeks 12 weeks	µCT scan Histology	It enables various bioactive molecules to be incorporated into printed CHA materials and provides a method of bioprinting biomaterials without compromising their natural properties.	Yes	Lin et al. (2016)
7	PLA-HA composite scaffolds (PLA 85% + HA 15%)	Rabbit tibial model	4-cm incision	NZW adult rabbits (N = 24, age: 6 months, 2.5 ± 0.2 kg)	4 weeks 8 weeks	µCT scan Histology	Generates vascularized tissue-engineered bone *in vivo*	Yes	Zhang et al. (2017)
8	MGPC (mMCS, GA, and PCL)	Rabbit femoral defects	Φ = 5 × 5 mm	NZW, 3 months old, 3–4 kg (N = 27)	4 weeks 8 weeks 12 weeks	Histology	Enhances osteogenesis and has great potential for bone regeneration	No	Zhang et al. (2018)
9	Fe^+3^ and Si^+4^ Doped β-TCP	Rat femoral defects	Intramedullary cortical defect	Male SD rats (N = 24), 3–3.5 kg in weight	4 weeks 8 weeks 12 weeks	Histomorphometry	Enhances osteogenesis and angiogenesis	No	Bose et al. (2018)
10	SiO2 and ZnO doped TCP	Rabbit tibial defect model	Critical-sized bone defects (length 0.7 cm and radius 0.3 cm)	New Zealand white male rabbits (2–2.5 kg)	8 weeks 16 weeks	Radiological	Enhances bone formation and in turn, leads to accelerated healing	Yes	Nandi et al. (2018)
11	3D Porous Bone Substitute Based on Calcium Phosphate	Rat femoral defects	Semi-cylindrical defect: length 0.7 cm and radius 0.3 cm)	Wistar rats (N = 12), aged 25–30 weeks	24 weeks	Histology	Better osteoconductive properties	No	Dubrov et al. (2019)
12	3DPT (3D-printed PEEK with Ti)	Rabbit tibia defect model	4 mm in diameter and 8 mm in length	male NZW rabbits (N = 6), 8-week-old, body weight 3.0 kg	4 weeks 8 weeks 12 weeks	µCT scan Histology	Overall enhancements of cell attachment, proliferation, differentiation, and bone regeneration	Yes	Jung et al. (2019)
13	Sodium alginate and CaCl2 + PEGDA, GelMA, and I-2959	Swine model tibial defects	-	Male Bama mini pigs (N = 6, 25 kg)	12 weeks	µCT scan Histology	Provides sufficient strength and stiffness until bone remolding	Yes	Li et al. (2021)
14	AKT Bioceramic Scaffolds with Hollow Channels	femur defects in rabbits	Critical size defects: 7 mm in diameter and 10 mm in length	Fifteen adult New Zealand rabbits (male, 10-month-old average)	12 weeks	µCT scan Histology	Improves the bioactivity of biomaterials for bone tissue engineering	Yes	Feng et al. (2021)
15	PLA and SDF-1 or BMP-7 immobilized in collagen type I	Rat femoral defect	diameter: 6 mm	Ten-week-old Wistar rats (N = 36)	4 weeks 8 weeks	µCT scan X-ray Histology	Capable of inducing bone regeneration in a critical size defect in rats.	No	Lauer et al. (2020)
16	PCL Scaffold Combined with Co-Axially Electrospun Vancomycin/Ceftazidime/Sheath-Core Nanofibers	Rabbit fracture model	Critical size bone defects: 10 mm in length	NZW rabbits (N = 15) (2–2.5 Kg)	3 months	Radiology Biomechanical evaluation	Facilitate bone healing by inducing bioactive membrane	No	Yu et al. (2020)
17	PLA scaffold with Biogel composed of gelatin and alginate	Rabbit tibial defect	Critical-size bone defect)	NZW rabbits	4 weeks	µCT scan, histology	3D PLA-Biogel-based scaffold adapted rhBMP-2 and MSCs with carrier PLA showed good biocompatibility and high possibility as an effective and satisfactory bone graft material	No full text	Han et al. (2020)
18	PTMC containing high ratios of TCP	Rabbit tibial defect	critical size bone defects: ∼8 mm circular defect	NZW rabbit, 5–6 months old (N = 10)	8 weeks 16 weeks	µCT scan, histology	These composites act as a next-generation synthetic bone substitute	Yes	Teotia et al. (2020)
19	Gene-activated implants based on OCP and plasmid DNA encoding *VEGFA*.	Segmental tibial defects in adult pigs	Length of 30 mm	Male pigs body weight (50 ± 2 kg, N = 4)	3 months 6 months	Histology	Effective approach to overcome current limitations in the production of personalized implants for critical size bone defect reconstruction	No	Bozo et al. (2020)
20	PCL scaffolds fibrin-based hydrogel, gelatin methacrylamide, fibrin and alginate	Rat femoral bone defects	Critical size defect: 5 mm mid-diaphyseal	Male Wistar Han rats, 12-week old (N = 27)	6 weeks 12 weeks	μCT scan Histology	Enhance the vascularisation and regeneration of large bone defects *in vivo*	Yes	Nulty et al. (2021)
21	PLA 100M^+β−TCP^	Rat femur window defect model	-	13–15 months old male SD rats	6 weeks	μCT X-ray Histology	Showed a positive biosafety profile and enhanced new bone formation	Yes	Fairag et al. (2021)
22	CpTi-P	Rat and Rabbit distal femur defects	3 mm diameter × 5 mm long	Male SD rats (280–300g) and NZW rabbit (3.5–4 kg)	5 weeks 7 weeks	μCT Histology	*in vitro* cytocompatibility and early stage *in vivo* osseointegration	No	Mitra et al. (2021)
23	BGS (SiO2: CaO: P2O5 = 35:50:15)	Rabbit femoral defect Model	1.0 or 1.5 cm segmental defect	NZW rabbit (N = 6)	2 weeks 4 weeks 8 weeks 12 weeks	X-ray Histology	Enhances *in vivo* Osteogenesis	No	Zhao et al. (2021)
24	HA/PLGA copolymer	Rabbit femoral defect Model	-	NZW rabbit	6 months	radiography and histology	Enhances *in vivo* Osteogenesis	No	Wu et al. (2021)
25	GelMA Scaffolds (15% w/v)	Rat femoral defect	diameter of 3 mm and a depth of 2 mm	6-week-old male SD rats	8 weeks	µCT scan Histology	Bone regeneration and repair of bone defect	Yes	Chai et al. (2022)
26	graphene-containing (1, 3, 5, 10 wt%), porous and oriented PCL scaffolds	Rabbit distal femur defect		NZW rabbit		Radiography Histology	Repairs osteochondral defect areas	No full text	Basal et al. (2022)
27	Gelatin/PCL membrane as a GBR construct	Canine tibia bone defects	5.8 mm (a depth of 2 mm, and a 5 mm gap)	Canines weighing (N = 4, 20–25 kg)	8 weeks	Histology Radiography	Increases bone density in comparison to the control group	No	Jamalpour et al. (2022)
28	hydroxyapatite (HA) scaffolds	rat tibial defect model							Chakraborty et al. (2022)
29	IONPs-labeled PCSCs-hydrogels	Rabbit femoral defect	5 mm	NZW rabbits (N = 12, 3.5 Kg)	12 weeks	μCT scans Histology	Enhances osteogenesis	Yes	Liao et al. (2022)
30	icariin-loaded Ti6Al4V reconstruction rod	Beagle dog femoral head necrosis model	Diameter of 5 mm and a length of about 30 mm	Beagle dog (N = 12)	3 weeks	μCT scans, MRI X-Ray, Histology	Facilitates osteogenesis and neovascularization, leading to effective osseointegration	Yes	Lei et al. (2023)
31	fibrin based bioinks and bioprinted PCL frameworks: fibrinogen, type A gelatin, HA and glycerol based bioink	Rat femoral defect	Critical size defect: 5 mm	12-week-old rats, Wistar Han rats	6 weeks 12 weeks	μCT scans Histology	Capable of supporting large bone defect regeneration	No	Pitacco et al. (2023)
32	PLGA (ratios of LA: GA-65:35 and 75:25) and blended with graphene nanoparticles	Rat segmental femoral bone defect	5 mm	12-week-old SD rats (N = 24, 22–24 g)	8 weeks	μCT scans X-ray Histology	Biocompatible, has no side effects, and enhances bone repair	No	Newby et al. (2023)
33	RP scaffold (cylindrical Si-CAOP scaffolds	Rat femoral segmental defect model	6 mm	six-month-old female Wistar rats (N = 80)	3 months 6 months	μCT scans Histology	Enhances bone regeneration and vascularization of critical size discontinuity bone defects	No	Knabe et al. (2023)
34	HA 3D-printed scaffolds with Gyroid-TPMS	Large animal model (sheep femur)		2-3-year-old sheep	4 weeks 26 weeks	X-ray Histology	Bone regeneration in view of clinical practice	No	Knabe et al. (2023)
35	PDA-β-TCP/PCL composite scaffolds	Rabbit distal femoral condyle defect	6 mm diameter and a 6 mm depth	Male NZW rabbits aged three Months (1.5–2 kg)	4 weeks 8 weeks	μCT scans X-ray Histology	PDA enhances both physicochemical and biological properties	Yes	Ho et al. (2022)
36	HA/β-TCP/SF	Rabbit tibia defect model		6-month-old NZW rabbits (N = 30, 2.5–3 Kg)	1 month 3 months	μCT scans X-ray Histology	Positive effects on bone formation *in situ*	Yes	Li et al. (2023)
37	GO-PCL scaffolds	Rabbit tibia defect model	2 cm rectilinear incision	6-month-old NZW rabbits	4 weeks 8 weeks	Histomorphometry	Improves biodegradability and wetting properties of PCL scaffolds	Yes	Alazab et al. (2023)

CRCL: cranial cruciate ligament, NZW: new zealand white, Ti: Titanium, OCP: octacalcium phosphate, BGS: bioactive glass scaffold, PCL: poly-ε-caprolactone, HA: hyaluronic acid, IONP: iron oxide nanoparticles.

**TABLE 2:** Methodological characteristics of the studies included in the meta-analysis for the repair and regeneration of bone.

Animal bone defect model	Duration of treatment	Outcome type	Control group			Experimental group			Included in meta-analysis	References
			Mean 0	SD0	N0	Mean1	SD1	N1		
Rabbit femoral defect	8 weeks	NBA (%)	20.3	2.63	6	31.85	6.67	6	Yes	Tarafder et al. (2015)
	12 weeks		47.55	8.73	6	59.35	3.79	6		
Rabbit femoral defect	6 weeks		3.22	0.48	10	8.01	0.72	10	No	Liu et al. (2016)
	12 weeks		8.03	0.77	10	15.37	0.86	10		
Rabbit femoral defect	8 weeks		0.49	0.34	5	14.89	1.12	5	Yes	Nandi et al. (2018)
	16 weeks		3.16	1.87	5	27.05	1.95	5		
Rabbit femoral defect	12 weeks		8.5	1.5	6	12.1	0.4	6	Yes	Feng et al. (2021)
Beagle dog femoral defect	3 weeks		33	4	4	42	4	4	Yes	Lei et al. (2023)
Rabbit tibial defect	4 weeks		16.8	1.53	15	43.8	4.38	15	Yes	Alazab et al. (2023)
	8 weeks		20.3	2.26	15	52.4	4.03	15		
	4 weeks		16.8	1.53	15	41.9	3.49	15		
	8 weeks		20.3	2.26	15	50.6	5.62	15		
Rabbit femoral defect	6 weeks	BV/TV (%)	4.208	0.693	10	10.58	0.85	10	Yes	Liu et al. (2016)
	12 weeks		9.59	0.97	10	18.49	0.947	10		
Rabbit femoral defect	4 weeks		10.18	1	12	15.752	1.258	12	Yes	Lin et al. (2016)
	8 weeks		14.96	2.33	12	26.7	1.26	12		
	12 weeks		23.65	1.45	12	37.6	2.58	12		
	4 weeks		10.18	1	12	14.88	0.25	12		
	8 weeks		14.96	2.33	12	25.83	1.96	12		
	12 weeks		23.65	1.45	12	37.86	2.02	12		
	4 weeks		10.18	1	12	11.68	1.32	12		
	8 weeks		14.96	2.33	12	24.08	1.64	12		
	12 weeks		23.65	1.45	12	32.01	1.58	12		
Rabbit tibial defect	4 weeks		20.4	0.67	6	36.17	1.01	6	No	Zhang et al. (2017)
	8 weeks		31.9	0.82	6	71.4	0.83	6		
Swine tibial defects	12 weeks		53.47	8.79	3	74.8	12.51	3	No	Li et al. (2021)
Rabbit femoral defect	12 weeks		14.2	11.4	6	20.9	4.5	6	Yes	Feng et al. (2021)
Rabbit tibial defect	16 weeks		8.5	0.7	5	9.9	1.5	5	No	Teotia et al. (2020)
Rat femoral defect	6 weeks		25.46	5.22	8	38.65	3.21	8	Yes	Fairag et al. (2021)
Rat femoral defect	8 weeks		11.92	2.26	3	35.41	5.12	3	No	Chai et al. (2022)
Rat femoral defect	12 weeks		10.1	1.27	4	32.3	7.2	4	Yes	Liao et al. (2022)
Beagle dog femoral defect	3 weeks		60.6	3.6	4	71	3	4	Yes	Lei et al. (2023)
Rabbit femoral defect	4 weeks		10.68	1.22	3	14.36	1.11	3	Yes	Ho et al. (2022)
	8 weeks		19.68	0.77	3	26.74	2.67	3		
	4 weeks		10.68	1.22	3	18.92	1.23	3		
	8 weeks		10.68	1.22	3	37.09	3.56	3		
Rabbit tibial defect	4 weeks	Tb. Th. (mm)	0.13	0.01	6	0.19	0.004	6		Zhang et al. (2017)
	8 weeks		0.27	0.01	6	0.32	0.01	6		
Swine tibial defects	12 weeks		0.4589	0.1332	3	0.7757	0.109	3		Li et al. (2021)
rat femur defect	6 weeks		0.16506	0.02549	8	0.22582	0.22582	8	Yes	Fairag et al. (2021)
Rat femoral defect	12 weeks		0.204	0.039	4	0.335	0	4	Yes	Liao et al. (2022)
Rat femoral defect	8 weeks		0.13	0.05	3	0.31	0.01	3		Chai et al. (2022)
Rabbit femoral defect	4 weeks		0.09	0.019	3	0.12	0.02	3	Yes	Ho et al. (2022)
	8 weeks		0.09	0.019	3	0.16	0.02	3		
	4 weeks		0.14	0.01	3	0.22	0.04	3		
	8 weeks		0.14	0.01	3	0.3	0.04	3		
Rabbit tibial defect	4 weeks	Tb. N. (mm)	1.2	0.19	6	2.01	0.03	6	No	Zhang et al. (2017)
	8 weeks		2.12	0.12	6	3.1	0.48	6		
Swine tibial defects	12 weeks		1.2139	0.2303	3	1.538	0.4477	3		Li et al. (2021)
Rat femoral defect	6 weeks		1.53	0.14	8	2.1	0.35	8		Fairag et al. (2021)
Rat femoral defect	8 weeks		1.46	0.017	3	3.85	1.05	3		Chai et al. (2022)
Rabbit tibial defect	4 weeks	Tb. S. (mm)	0.76	0.01	6	0.5	0.06	6		Zhang et al. (2017)
	8 weeks		0.45	0.07	6	0.26	0.03	6		
Swine tibial defects	12 weeks		0.7148	0.1863	3	0.3866	0.1363	3		Li et al. (2021)
rat femur window defect model	6 weeks		0.74712	0.14279	8	0.46005	0.075	8		Fairag et al. (2021)
Rat femoral defect	8 weeks		0.86	0.086	3	0.24	0.034	3		Chai et al. (2022)
Rabbit tibial defect	4 weeks	BV (%)	140	16	6	149	13	6	Yes	Jung et al. (2019)
	8 weeks		166	7	6	180	13	6		
	12 weeks		203	12	6	217	27	6		
Rabbit femoral defect	6 weeks		411	84	8	685	53	8	No	Fairag et al. (2021)
Rabbit tibial defect	4 weeks		34.7	2.36	10	37.69	3.5	10	Yes	Li et al. (2023)
	12 weeks		66.27	2.93	10	75.93	3.26	10		

NBA: new bone area, Tb. Th.: trabecular thickness, Tb. N.: trabecular number, Tb. S.: trabecular separation, BV: bone volume.

**TABLE 3:** Data from pooled and subgroup analysis summarized for the studied parameters.

Parameters	Groups	Femoral or tibial bone defects (animal species)	Test of heterogeneity			Test model	Types of association				Significance
			Q	P	I^2^ (%)		SDM	Lower limit	Upper limit	*p*-Value	
BV/TV	Control vs. experimental group	Femoral defect (rabbits, rats, beagle dogs)	181.661	0.000	89.990	Random	4.545	3.383	5.707	0.000	Significant
BV/TV	Control vs. experimental group	Femoral defect (Rabbit)	181.642	0.000	90.090	Random	4.736	3.443	6.029	0.000	Significant
BV/TV	Control vs. experimental group	Tibial defect (Rabbit)	41.953	0.000	95.233	Random	20.024	0.738	39.309	0.042	Significant
NBA	Control vs. experimental group	Femoral defect (rabbits and beagle dogs)	15.619	0.008	67.987	Random	2.252	1.138	3.365	0.000	Significant
NBA	Control vs. experimental group	Femoral defect (Rabbit)	15.475	0.004	74.152	Random	2.282	0.958	3.606	0.001	Significant
NBA	Control vs. experimental group	Tibial defect (Rabbit)	10.506	0.062	52.408	Random	9.219	7.475	10.964	0.000	Significant
Tb. Th	Control vs. experimental group	Femoral defect (Rabbit and Rat)	5.427	0.3666	7.82	Fixed	2.401	1.594	3.208	0.000	Significant
Tb. Th	Control vs. experimental group	Femoral defect (Rabbit)	5.427	0.246	26.292	Fixed	2.514	1.376	3.653	0.000	Significant
BV	Control vs. experimental group	Femoral defect (Rabbit)	9.576	0.048	58.229	Random	1.314	0.352	2.276	0.007	Significant

### 3.2 Study characteristics

**FIGURE 2:**
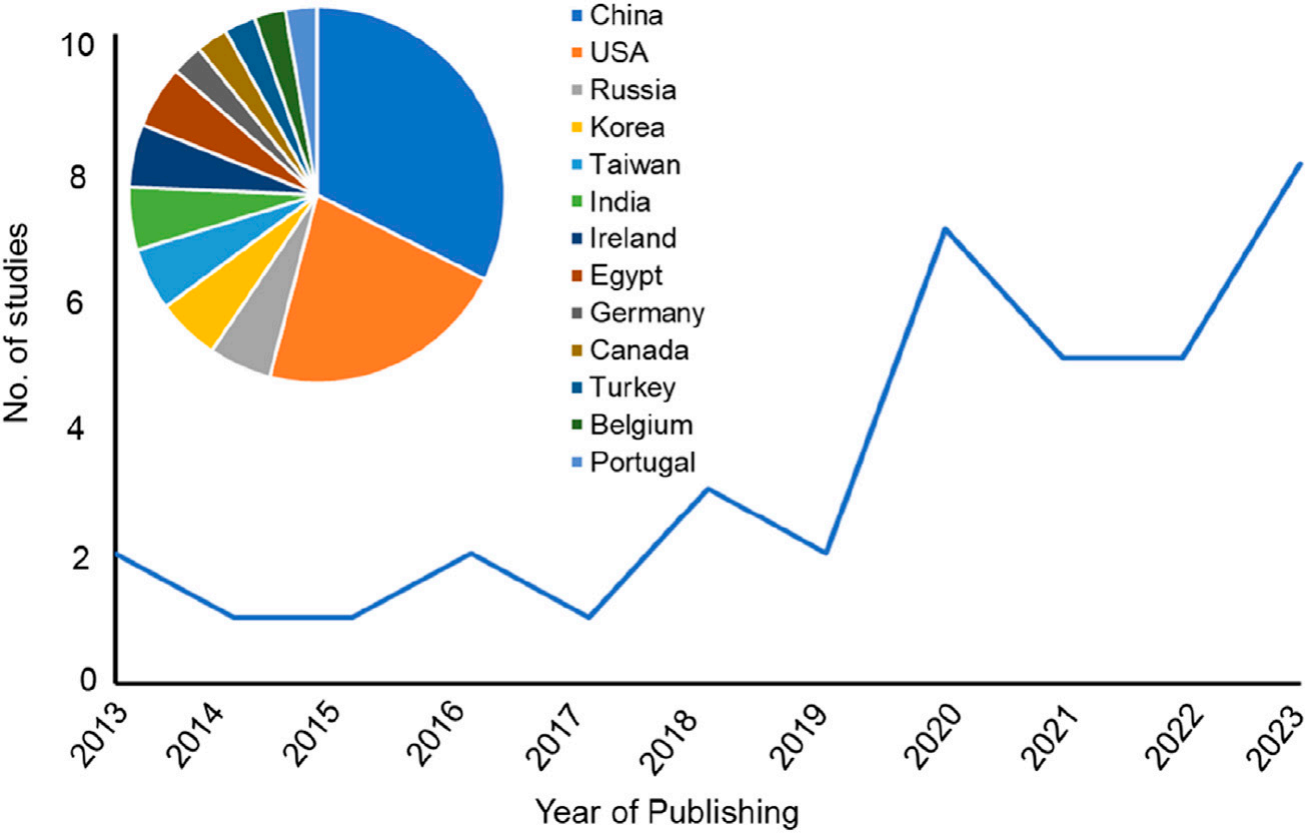
Line chart representing the number of the published studies included in the systematic review sorted by the year of publishing. Insert graph for a pie chart representing the country affiliations of all included papers in this systematic review.

The research articles included in this systematic review were published from January 2013- October 2023, focusing on the advancement of 3D-printed scaffolds for the repair of femoral and tibial defects in animal models. Indeed, 3D-printed scaffolds have emerged as a promising alternative for bone regeneration. Based on the qualitative data as shown in Supplementary Table S1, it is evident that a total of 37 articles were published over the last 10 years, a mean of 3.7 articles per year. Research groups in the USA and China predominantly initiated these studies. Within the past 5 years (2018–2023), the field was rapidly growing with 30 published articles dominated by research groups in the USA and China (Figure 2).

Within the included studies, 3D-printed composite scaffolds were employed in addressing femoral and tibial defects across five distinct animal models. Specifically, 20 studies were conducted in New Zealand White (NZW) rabbits, 11 studies involved rats (comprising 5 studies with the Wistar strain and 6 studies with Sprague-Dawley rats), 3 studies utilized dogs (2 studies involving beagle dogs and 1 study with canines), 1 study involved sheep, and 2 studies employed pigs. The predominant focus of the studies centered on rabbits, examining the impact of a variety of printed biomaterials, scaffolds, and templates with different combinations and ratios (Supplementary Table S1). More than half of the included studies were found to combine printed templates with cells, growth factors, or both. Histology or micro-computed tomography (µCT) or both emerged as the most common methods to assess the repair of femoral and tibial bone defects. Importantly, critical-sized bone defects (CSD) are not expected to heal spontaneously within the lifetime of the animal. In this systematic study, all the defects made in animals were performed in the femur or tibia. Still, only 9 could be considered as CSD, performed in the femur of rabbit (N = 4) and rat (N = 3) and tibia of rabbit (N = 2) respectively.

### 3.3 Effects of 3D printed composite scaffolds on BV/TV of femoral and tibial defects in animal models

In our current study, we conducted a meta-analysis that was stratified based on two key factors: bone defects (specifically femoral or tibial) and animal models. This approach was taken to prevent any potential bias in the results stemming from differences in methodology and species used across various studies included in our analysis.

**FIGURE 3:**
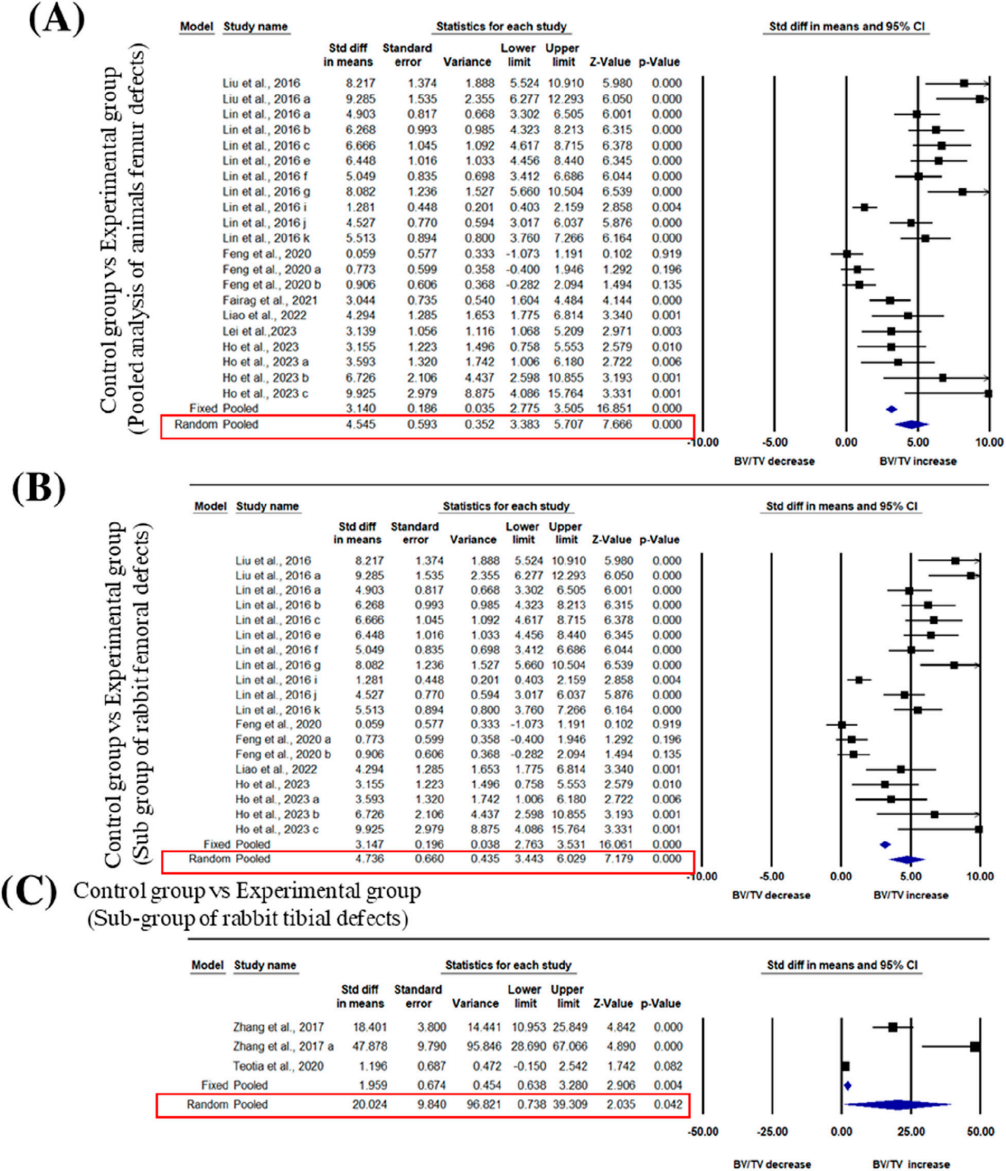
Composite scaffolds increase BV/TV of femur and tibial defects of animal models. **(A)** Forest plot of a pooled analysis of animal models of BV/TV of femoral defects **(B)** Forest plot of a subgroup of rabbit femur defects **(C)** Forest plot of a subgroup of rabbit tibial defects.

We pooled data from the studies that used 3D bioprinted composite scaffolds to determine the overall effect of fabrication in bone tissue engineering on a BV/TV of the femur and tibia of animal models. Six studies were included in the meta-analysis for femoral defects of BV/TV (Liu et al., 2016; Lin et al., 2016; Feng et al., 2021; Fairag et al., 2021; Chai et al., 2022; Lei et al., 2023; Ho et al., 2022). The pooled analysis was performed using a random-effect model which showed a significant increase in BV/TV of the femur after implantation of fabricated 3D printed composite scaffolds in animal models (rabbits, rats, beagle dogs) compared with control groups (SDM = 4.545, 95% CI = 3.383 to 5.707, *p* = 0.000) (Figure 3A). The funnel plots did not demonstrate apparent asymmetry for BV/TV (Supplementary Figure S1A) and the heterogeneity among studies was significant (*p* = 0.000, I^2^ = 89.990%, Q = 181.661).

A subgroup analysis was performed to analyze the effect of implantation of 3D printed scaffolds in rabbit femur. This analysis showed that 3D printed composite scaffolds significantly increased BV/TV of rabbit femur (SDM = 4.736, 95% CI = 3.443 to 6.029, *p* = 0.000) (Figure 3B). The heterogeneity among studies was relatively high (I^2^ = 90.090, Q = 181.642, *p* = 0.000) and funnel plots did not demonstrate apparent asymmetry for BV/TV of rabbit femur (Supplementary Figure S1B). In addition to that, another subgroup of rabbit tibial defect showed that implantation of 3D printed composite scaffolds significantly increased BV/TV of rabbit tibia in comparisons to control with significant heterogeneity among studies (SDM = 20.024, 95% CI = 0.738 to 39.309, *p* = 0.042 and I^2^ = 95.233, Q = 41.953, *p* = 0.000) (Figure 3C). The funnel plots also did not demonstrate apparent asymmetry for BV/TV of rabbit tibia (Supplementary Figure S1C).

### 3.4 Effects of 3D bio-printed composite scaffolds on NBA of femoral and tibial defects in animal models

**FIGURE 4:**
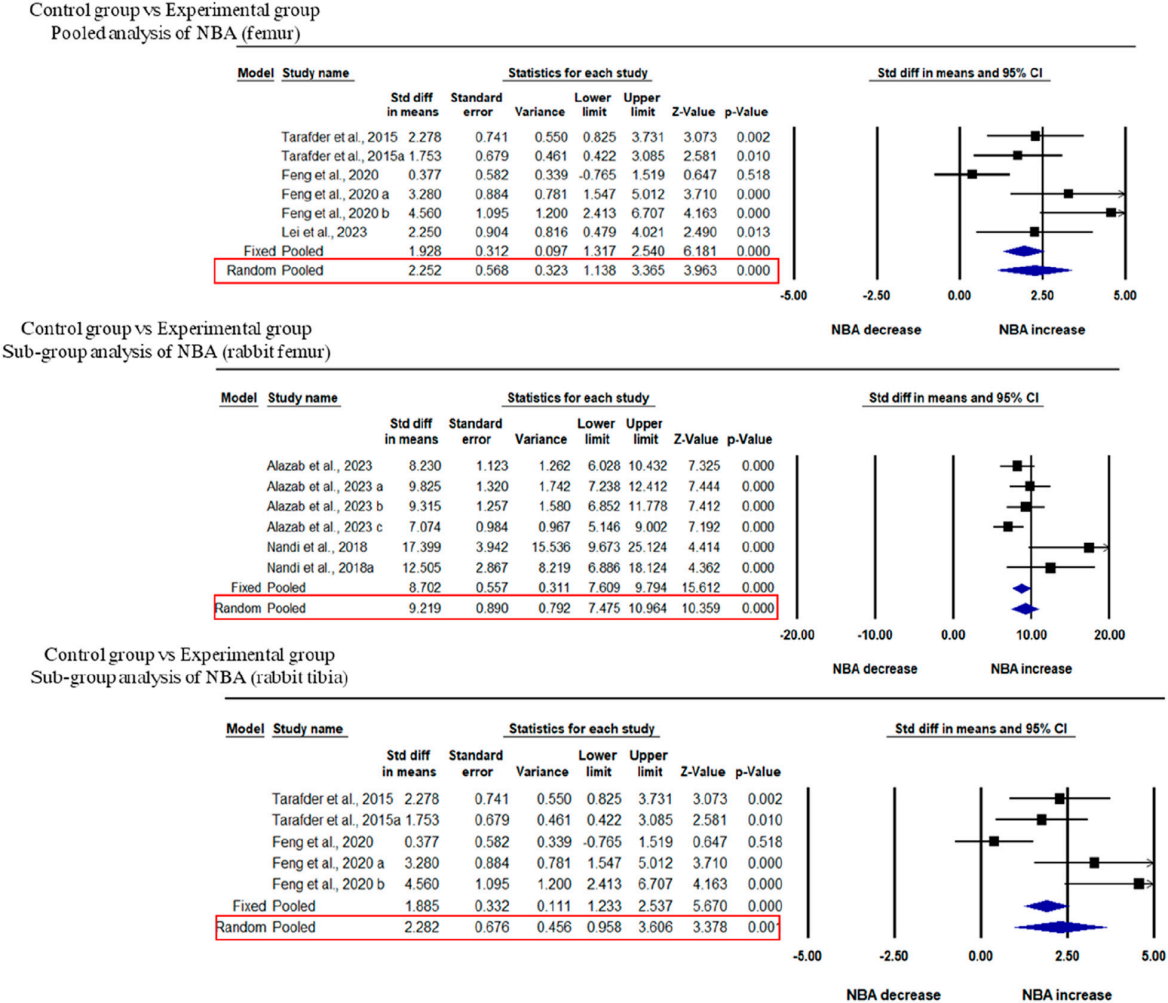
Composite scaffolds increase NBA of femur and tibial defects of animal models. **(A)** Forest plot of a pooled analysis of animal models of NBA of femoral defects **(B)** Forest plot of a subgroup of rabbit femur defects **(C)** Forest plot of a subgroup of rabbit tibial defects.

Three studies were included in the meta-analysis for NBA regeneration in animal models of femoral defects. The pooled analysis was performed using a random-effect model which showed a significant increase in the regeneration of NBA of the femur after implantation of fabricated 3D printed composite scaffolds in animal models (rabbits, and beagle dogs) compared with control groups (SDM = 2.252, 95% CI = 1.138 to 3.365, *p* = 0.000) (Figure 4A) and significant heterogeneity was observed among studies (I^2^ = 67.987, Q = 15.619, *p* = 0.008). A subgroup analysis was performed to analyze the effect of implantation of 3D printed scaffolds in rabbit femur. This analysis showed that 3D printed composite scaffolds significantly increased NBA of rabbit femur (SDM = 2.282, 95% CI = 0.958 to 3.606, *p* = 0.001) (Figure 4B). The heterogeneity among studies was relatively high (I^2^ = 74.152, Q = 15.475, *p* = 0.004). Another subgroup of rabbit tibia showed that 3D printed composite scaffolds increased NBA (SDM = 9.219, 95% CI = 7.475 to 10.964, *p* = 0.000) (Figure 4C). The heterogeneity among studies was relatively high (I^2^ = 52.408, Q = 10.506, *p* = 0.062).

### 3.5 Effects of 3D bioprinted composite scaffolds on Tb. Th. and BV of tibial defects in rabbits

**FIGURE 5:**
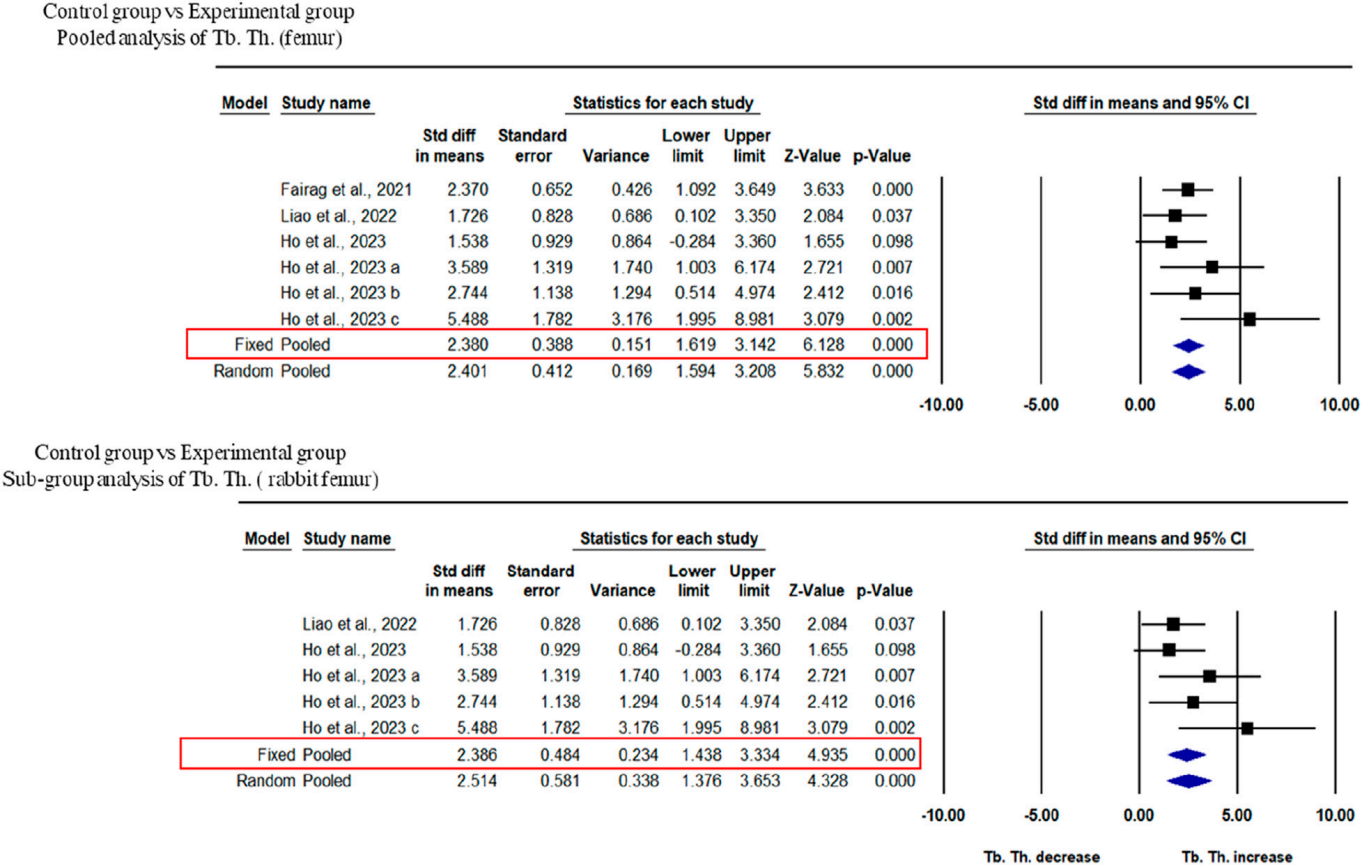
Composite scaffolds increase Tb. Th. of femur and tibial defects of animal models. **(A)** Forest plot of a pooled analysis of animal models of Tb. Th. of femoral defects **(B)** Forest plot of a subgroup of femoral defects of rabbit.

Three studies were included for the analysis of trabecular thickness. A pooled analysis of femoral defects of animal models (rabbit and rat) showed that 3D-printed composite scaffolds significantly increased Tb. Th. of the femur (SDM = 2.401, 95% CI = 1.594 to 3.208, *p* = 0.000) (Figure 5A). The heterogeneity among studies was relatively low (I^2^ = 7.872, Q = 5.427, *p* = 0.366). A subgroup analysis of rabbit femoral models also showed a significant increase in Tb. Th. after the implantation of 3D printed scaffolds (SDM = 2.514, 95% CI = 1.376 to 3.653, *p* = 0.000 and I^2^ = 26.292, Q = 5.427, *p* = 0.246) (Figure 5B).

**FIGURE 6:**
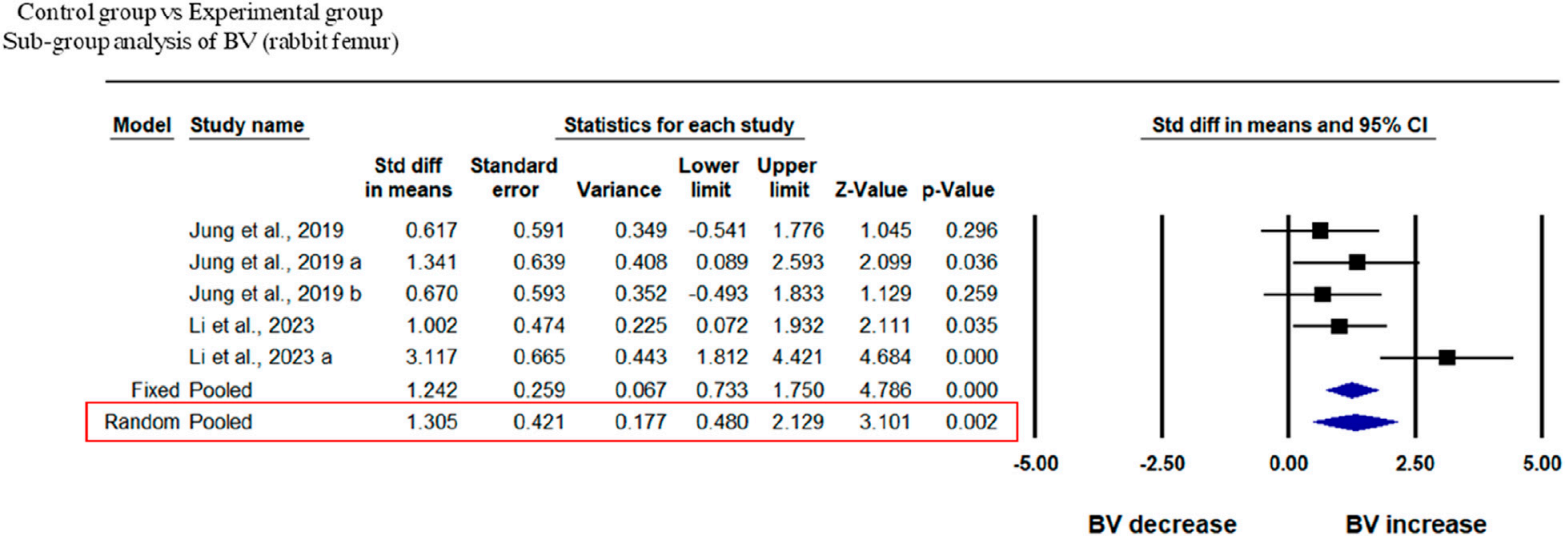
Composite scaffolds increase BV of rabbit tibial defects. Forest plot of BV of rabbit tibia.

Two studies at different time points were included in this study to analyze the BV of rabbit femur after implantation of fabricated 3D printed composite scaffolds in comparison with control groups. This analysis showed that 3D-printed composite scaffolds significantly increased the BV of rabbit tibia (SDM = 1.314, 95% CI = 0.352 to 2.276, *p* = 0.007) (Figure 6). The heterogeneity among studies was relatively high (I^2^ = 58.229, Q = 9.576, *p* = 0.048).

### 3.6 Publication bias

We undertook publication bias analysis qualitatively based on asymmetry in the funnel plot and quantitatively based on Egger’s intercept test. We conclude that the estimated effects are free of bias for most parameters; however, for the remainder, we have provided unbiased estimates using the trim and fill method.

### 3.7 Sensitivity analysis

The sensitivity analysis was performed with the exclusion of one study at a time. No study was found to be sensitive enough to change the conclusion.

## 4 Discussion

### 4.1 Summary of results

Tissue engineering presents a diverse range of innovative approaches to regenerate bone tissue, with the fabrication of 3D porous scaffolds using biodegradable materials emerging as a prominent method. This strategy effectively mimics key characteristics of natural bone tissue, contributing to innovative solutions for bone regeneration (Shin et al., 2003). The major forms of 3D printing used for bone-tissue engineering materials include extrusion, stereolithography, selective laser sintering, and inkjet printing. This systematic review and meta-analysis aimed to evaluate the possible use of 3D-printed composite scaffolds in bone tissue engineering, especially in the femur and tibial defects of animal models. Femur and tibia of animals are of significant interest due to their load-bearing roles, anatomical similarities to human bones, high incidence of clinically relevant fractures, and utility as robust models for testing and validation. These bones often present critical-sized defects that cannot heal on their own, making them ideal candidates for tissue-engineered solutions that can fill and repair large gaps. The composite scaffolds provide a 3D environment for cell seeding and proliferation as well as repair of bone defects in animals while ensuring mechanical strength during the process of bone regeneration.

### 4.2 The role of scaffolds architecture in bone tissue engineering

The internal architecture of scaffolds is recognized as a pivotal factor in tissue engineering, influencing both mechanical and biological properties. Scaffolds featuring highly interconnected 3D pores offer advantages by promoting cell adhesion, facilitating mechanical interlocking between the host tissue and scaffold through bone ingrowth, and supporting the transport of nutrients and metabolic waste. However, scaffolds need to possess adequate strength to withstand *in vivo* stresses at the application site until the biodegradable scaffold matrix is replaced by newly formed bone through the process of bone regeneration. The critical parameters investigated in this study include pore interconnection, microporosity, macroporosity, overall porosity, pore size, and pore shape within the 3D bio-printed composite scaffold which play a critical role in the joining of bone-implant boundary (Otsuki et al., 2006; Van Bael et al., 2012). A diverse range of pore sizes is applicable for bone regeneration, where macroporosity generally supports osteogenesis, and microporosity enhances surface area for protein adsorption, providing attachment points for bone-forming cells. Studies suggest an optimal pore size ranging from 200 to 500 µM, with a minimum size of 100 μm to avoid non-mineralized osteoid or fibrous tissue formation, which could limit oxygen and nutrient diffusion throughout the scaffolds (Bohner et al., 2005). Maintaining pores above 300 μm is recommended to facilitate cell proliferation and enhance neovascularization. Pore interconnectivity positively influences the rate and depth of bone deposition, improving nutrient and oxygen supply to the scaffold’s inner part and facilitating cell infiltration. AKT-H-N scaffolds featuring hollow channels and a micro-nano surface were successfully created by 3D printing approach coupled with hydrothermal treatment, leading to a slight improvement in mechanical strength (Feng et al., 2021). The AKT-H-N scaffolds demonstrated enhanced attachment and proliferation of BMSCs *in vitro*, with the hollow channels proving capable of accommodating more cells than solid scaffolds. The scaffold, characterized by its micro-nano surface and hollow channels, overcomes limitations associated with traditional scaffolds and exhibits synergistic effects in promoting bone regeneration.

### 4.3 Role of 3D printed calcium phosphates in femoral and tibial defects of animal models

Various scaffolds have been developed and tested in animal models to facilitate bone tissue regeneration (Giron et al., 2021). CaP bioceramics have been widely used in bone tissue engineering due to their outstanding bioactivity, osteoconductivity, and similarity in composition to natural bone. Notably, CaPs, such as β-TCP stand out due to their bioresorbable nature, allowing for gradual degradation over time with concurrent host tissue ingrowth, making it suitable for diverse applications. Besides biocompatibility, three crucial factors contribute to the success of scaffold material for bone implants: osteoinduction, osseointegration, and osteoconduction (Habibovic et al., 2005). β-TCP is recognized for its osteoconductive properties; however, it inherently lacks osteoinductive capabilities. Substantial efforts have been directed toward enhancing this quality through the incorporation of pharmaceuticals and biologics (Matsumoto et al., 2007; Yoshinari et al., 2002; Lan Levengood et al., 2010). Among 37 studies, only nine studies specify employing a 3D-printed β-TCP template. These templates involve different combinations of biomaterials and are applied in addressing femoral and tibial bone defects within animal models.

3D printed akermanite scaffolds were introduced by a research group from China at Zhejiang University, Hangzhou, China in 2016 with desirable interconnected pores and appreciable compressive strength (>70 MPa). The akermanite scaffolds were fabricated and applied in critical size defects (6 × 6 mm Ø) in male NZW rabbits for its osteogenesis effect and mechanical evolution at 6 and 12 weeks, in comparison with the clinically available β-TCP material which is fabricated by the same 3D printing technique (Liu et al., 2016). The akermanite scaffolds enhance tissue regeneration and repair of load-bearing bone defects. 3D-printed akermanite scaffolds (Ca2MgSi2O7), utilizing the well-established osteoinductive properties and predictable degradation rate of akermanite, represent a significant advancement in bone graft technology, manufactured by the widely-adopted ceramic ink writing technique. Notably, their degradation rate surpasses that of β-TCP counterparts. An intriguing aspect of the akermanite scaffolds is the unique release of Si and Mg. Furthermore, the concentration of calcium (Ca) release from akermanite scaffolds is more than three times higher than that from β-TCP scaffolds (Wu and Chang, 2007). This distinctive feature contributes to the overall efficacy of the akermanite scaffolds in promoting bone regeneration for load bearing bone defects (Liu et al., 2016).

#### 4.3.1 Role of added dopant in to 3D printed β-TCP

The addition of dopant into β-TCP such as silicon, zinc, strontium, magnesium, and metal oxide such as SiO_2_/ZnO not only allow for the customization of strength but also augments the biological response both *in vitro* and *in vivo* (Bohner, 2009; Bose et al., 2011; Fielding and Bose, 2013). In earlier attempts, β-TCP (particle size around 550 nm and specific average surface area of 10–50 m^2^g^−1^) scaffolds were fabricated through the direct inkjet 3D printing method and used in the absence of cells by a research group from the USA in 2013 (Fielding and Bose, 2013). Subsequently, these fabricated scaffolds, including both the β-TCP template and SiO_2_/ZnO doped β-TCP, were implanted into the femoral defect model of SD rats (diameter 2.5 mm) and evaluated using histology and histomorphometry over 16 weeks for osteogenic properties. The addition of SiO2 and ZnO was found to provide robust osteoinductive capabilities to β-TCP bone replacement materials. Pure β-TCP and SiO2/ZnO doped β-TCP exhibited complete mineralized bone infiltration into the micropores at the implant interface after 6 weeks at the defect site of the femur in SD rats. However, doped β-TCP displayed additional new bone tissue growth in macropores and increased new bone formation compared to pure β-TCP. By week 12, both samples demonstrated extensive tissue integration, and SiO_2_/ZnO doped β-TCP showing significantly higher bone formation (Fielding and Bose, 2013). The inclusion of ZnO and SiO_2_ in the CaP scaffolds has successfully established an efficient delivery system for Zn^2+^ (linked with osteoclastic bone resorption) and Si^4+^ (linked with increased bone mineralization and bone growth) (Hie et al., 2011; Matsko et al., 2011; Hing et al., 2006).

Likewise, Fe^+3^ and Si^+4^ doped TCP scaffolds were fabricated and implanted into a rat distal femur model for the duration of 4, 8, and 12 weeks, leading to enhanced new bone formation and neovascularization (Bose et al., 2018). The addition of Fe and Fe-Si has shown to improve the densification of TCP, as evidenced by average volume shrinkages of 17.89%, 16.76%, and 10.00% for Fe-doped, Fe-Si-doped, and pure samples, respectively (Bose et al., 2018). Pure TCP, as well as Fe and Fe-Si-doped TCP, exhibited compressive strengths of 4.9 ± 0.7 MPa, 17.9 ± 1.3 MPa, and 19.8 ± 2.4 MPa respectively (Bose et al., 2018). Additionally, in a rabbit tibia model, the incorporation of SiO_2_/ZnO into TCP has shown an enhanced capacity to promote early bone formation and improve implant stability compared to using pure TCP alone. Radiographs suggest a slower degradation of SiO_2_/ZnO doped TCP, indicating increased scaffold stability within the body, thereby providing longer-term support for bone regeneration (Nandi et al., 2018). In conclusion, the added features were identified as key contributors to enhancing the ability of composite printed β-TCP templates to facilitate the repair of femur and tibia, surpassing the performance of β-TCP templates alone.

#### 4.3.2 Role of added dopants and microwave sintering of 3D printed β-TCP

The most critical factor for a scaffold is to provide adequate mechanical support for bone tissue engineering. Microwave sintering of ceramics have been extensively employed to enhance mechanical properties (Tarafder et al., 2013). The microwave sintering (volumetric heating) of β-TCP scaffolds significantly impacts porosity, mechanical strength and biological responses. Compared to conventional sintering, microwave sintering results in higher densification due to higher shrinkage, thus reducing total porosity (42%), and enhancing mechanical strength (10.95 ± 1.28 MPa) after sintering. These scaffolds exhibit excellent *in vitro* and *in vivo* biocompatibility, and enhances osteogenesis with smaller pore size both *in vitro* and *in vivo* (Tarafder et al., 2013). Generally, interconnected macro pores exceeding 300 μm are favorable for osteogenesis and vascularization, while the minimum effective recommended pore diameter for osteogenesis is 100 μm. The given study utilized a sintered pore size of −150 μm, suitable for *in vivo* osteogenesis and tissue in growth (Tarafder et al., 2013). *In vitro* studies indicated an increase in cell density with a decrease in pore size and *in vivo* studies demonstrated that both micro and macro pores actively facilitated osteogenesis after 2 weeks in femur model of male SD rats (Tarafder et al., 2013). In line with these findings, the incorporation of SrO and MgO as a dopants in microwave-sintered 3D printed β-TCP scaffolds enhances mechanical strength (compressive strength 12.01 ± 1.56 MPa; designed pore size 500 µM), osteogenesis and vasculogenesis in the early stages, crucial for accelerated wound healing in rabbit femoral condyle defect model (Tarafder et al., 2015; Bose et al., 2018; Yang et al., 2011). The presence of Sr^2+^ and Mg^2+^, facilitates effective cell penetration and provides pathways for nutrient transport through vascularization in newly formed bone tissue.

Moreover, microwave sintering and fabrication of 3D printed β-TCP with other biomaterials such as PLA/polycaprolactone (PCL), polydopamine (PDA)/PCL, and hydroxyapatite (HA)/silk fibroin (SF) have been shown to enhance bone formation and in turn leading to accelerated healing (Fairag et al., 2021; Ho et al., 2022; Li et al., 2023). Several other bioceramic materials have been utilized, including a 3D porous bone substitute made from CaP (n = 1), a customized bioceramic cage (n = 1), poly (trimethylene carbonate) (PTMC) with elevated levels of bioactive ceramics (n = 1), and gene activated octacalcium phosphate (OCP) (n = 1) to enhance bone formation in the femur and tibial defects of animal models (Teotia et al., 2020; Dubrov et al., 2019; Castilho et al., 2014; Bozo et al., 2020).

#### 4.3.3 Role of 3D printed HA composite scaffolds in femoral and tibial defects of animal models

HA is another main bioceramic chosen for the synthesizing composites (n = 6), being used as powders mixed in different ratios with the polymeric material. 3D-printed HA scaffolds have been used for the repair of femoral and tibial defects of animal models from 2016 onwards. One study used the printed HA templates in the tibial defect model of rats (Chakraborty et al., 2022) while another study used 3D-printed collagen−HA (CHA) scaffolds which were implanted into rabbit femoral condyle defect model (Φ = 5 mm, L > 10 mm) for 12 weeks and bone repair was evaluated using µCT scan and histology. Interestingly, HA and collagen stand out as key components in bone composition (Lin et al., 2016). HA offers exceptional biocompatibility, osteoconductivity, and bioactivity, while collagen boasts biocompatibility, biodegradability, and osteoinductivity (Swetha et al., 2010). A composite CHA scaffold (collagen and HA ratio: 1:2 w/w) was printed using robocasting approach at 4°C (Lin et al., 2016). CHA scaffolds induce the proliferation of BMSCs and promote osteogenesis both *in vitro* and *in vivo* (Lin et al., 2016). CHA scaffolds have been demonstrated to combine the advantages of the mechanical strength of ceramics with the biological advantages of collagen (Swetha et al., 2010; Wahl and Czernuszka, 2006). Although one drawback of this study is the relatively modest mechanical strength of CHA materials, their application appears well-suited for the repair of low-load-bearing bone defects or cancellous bone defects.

Polymer-based composite scaffolds have been fabricated using 3D bioprinting, incorporating precise pore sizes, morphologies, porosity, and interconnected pores which enhances cell ingrowth and facilitates nutrient delivery. Another group from China in 2017 used PLA-HA composite scaffolds that were seeded with BMSCs crossed with a vascular bundle and transplanted into rabbit tibial periosteum for 8 weeks to analyze neovascularisation and bone tissues using µCT and histological examinations (Zhang et al., 2017). Addition of PLA to HA have been shown to enhance the regeneration of new bone and good biocompatibility and bioactivity *in vitro* (Zhang et al., 2016). The *in vivo* bioreactor assessment of a 3D-printed PLA-HA construct demonstrated notable osteogenic capability and osteoinductive activity, ultimately enhancing bone formation (Zhang et al., 2018). Few other studies used 3D printed HA scaffolds with Gyroid-Triply periodic minimal surface (TPMS) in clinically relevant large animal models (sheep femur) and HA/β-TCP/SF composite scaffold in tibial defect model of rabbit to enhance osteogenesis, analyzed using histology and X-ray (Li et al., 2023; Bouakaz et al., 2023).

### 4.4 Role of 3D-Printed PLA, PLGA and PCL in femoral and tibial defects of animal models

PLA is a biocompatible and degradable polymer derived from lactic acid which can be easily fabricated into porous scaffolds and used for synthesizing composite scaffolds. In recent years, PLA-HA scaffolds have proven effective in the regeneration of new bone (Zhang et al., 2017). Notably, large segmental defects typically manifest as critical bone damage, often characterized by a circumferential loss surpassing 50% or a length above 2 cm in adult patients (Nauth et al., 2011). Lauer et al. addressed CSD (6 mm) by 3D printing a PLA cylinder matching the dimensions of a rat femur defect. This PLA cylinder, incorporating type I collagen and immobilized SDF-1 or BMP-7 within the collagen matrix, was implanted into the rat femur defect (Lauer et al., 2020). After 8 weeks, bone regeneration analysis was confirmed by µCT and histological staining methods which showed the osteoinductive properties of this composite scaffold (Lauer et al., 2020; Bierbaum et al., 2012; Sarker et al., 2015). This study validates the osteoinductive nature of a novel, cell-free biomaterial fabricated through 3D bioprinting, opening up new possibilities for its utilization in the realm of bone tissue regeneration (Lauer et al., 2020).

Furthermore, 3D printed PLA combined with recombinant human bone morphogenetic protein-2 (rhBMP-2) and/or mesenchymal stem cells (MSCs) with Biogel composed of gelatin and alginate were investigated for bone regeneration both *in vitro* and *in vivo* studies (Han et al., 2020). *In vitro* studies revealed that PLA scaffold filled with both BMP-2 and MSCs loaded on Biogel (P-BG-B2-M) increased osteogenesis. After 4 weeks post-operation of NZW rabbits, µCT analysis revealed that within the tibial defect site, the P-BG-B2 group had significantly higher percent bone volume (BV/TV) than the PLA and P-BG-M groups. Outside the defect site, the P-BG-B2-M group showed significantly higher BV/TV than the PLA group (*p* < 0.05) (Han et al., 2020). A scaffold P-BG-B2-M displayed excellent biocompatibility and enhanced bone regeneration in critical-size bone defect in rabbit tibia. It holds promise as a highly effective bone graft material.

PLGA and PCL are the two most common resorbable polymers. PLGA, known for its non-toxicity to tissues and efficacy as a drug release carrier, exhibits minimal inflammatory response during degradation, yielding biocompatible end products. Numerous studies indicate that PLGA serves as an effective carrier for bone morphogenetic protein (BMP), thereby promoting enhanced bone healing. Calcium-deficient hydroxyapatite (CDHA)/PLGA bilayer scaffold demonstrated successful fabrication using 3D printing, achieving a favorable combination of both components. The study encompassed *in vitro* assessment of degradation, cytotoxicity, and cell proliferation along with *in vivo* evaluation of surgical safety, biodegradation, and osteogenic capacity using a rabbit femur cortical bone defect model (Wu et al., 2021). In conclusion, the CDHA/PLGA bilayer scaffold exhibited excellent biocompatibility without cytotoxic effects and holds potential for diverse clinical applications in bone repair through 3D-printing fabrication (Wu et al., 2021).

PCL shares similar advantages with PLGA but distinguishes itself by exhibiting a prolonged degradation time. This feature positions PCL as an excellent reservoir for preserving bone grafts until the completion of the bone healing process. Yu et al. (2020) innovatively devised a modified Masquelet procedure to address segmental bone defects. Their approach involved the utilization of a 3D-printed PCL scaffold combined with co-axially electrospun nanofibers containing PLGA, vancomycin, ceftazidime, and BMP-2 in a critical rabbit femoral bone defect model (Sabzevari et al., 2023). This method demonstrated the ability to maintain elevated and sustained concentrations of antibiotics and BMP-2, ultimately promoting effective bone healing (Yu et al., 2020). Moreover, PCL scaffolds containing different weights of graphene enhance bone regeneration in a large osteochondral defect in a rabbit model (Basal et al., 2022). Likewise, graphene oxide (GO) improved the physical and biological properties of PCL scaffolds and significantly enhanced new bone regeneration (Alazab et al., 2023). Finally, Newby et al. (2023). Implanted a novel 3D-printed composite scaffold with hMSCs, made of PLGA and graphene, resulting in a notable enhancement in bone mineralization within a rat segmental femoral bone defect.

### 4.5 Role of degradability of 3D-Printed scaffolds

An essential factor in bone scaffold manufacturing is the biodegradability of the material. Addressing the challenges posed by non-biodegradable implants, a study detailed the application of a custom bioceramic cage in treating a large domestic dog with a CrCL-deficient stifle using a modified TTA surgical technique. The cage exhibited an overall porosity of 59.2% with pore sizes measuring 845 μm. The outcome was a complete restoration of the dog’s limb function, free from lameness or adverse complications. Additionally, there was an enhancement in local biocompatibility and osteoconductivity (Castilho et al., 2014). Moreover, Li et al. investigated the feasibility of repairing sizable segmental bone defects in large living animal models, specifically swine, using *in situ* 3D bio-printing technology. This method, employing a robotic manipulator to control the extrusion-based layer-by-layer construction of a photopolymerization bio-ink, utilized a combination of alginate, PEGDA, and GelMA. This synergistic blend achieved an optimal degradation rate along with enhanced mechanical properties. The distinctive double network structure of this combination ensures the bio-inks stretchability while providing ample strength and stiffness throughout the bone remodeling process.

### 4.6 Role of 3D-printed bioactive glasses (BG) and silicate-based templates

Bioactive glass is a crucial biomaterial known for its strong biocompatibility, histocompatibility, cell compatibility, and osteoinductivity (Martelli et al., 2023). Utilizing the 3-D printing technique, bioactive glass porous scaffolds (BGS)-were fabricated, demonstrating the excellent capability of apatite formation. *In vitro* experiments revealed that the BGS has a good ability for the adhesion and proliferation of mouse bone marrow stromal cells (BMSCs) (Martelli et al., 2023). In a rabbit model with large bone defects, 3D-printed BGS significantly enhanced bone defect reconstruction, as evidenced by X-ray imaging at 2, 4, 8, 12, and 18 weeks post-surgery (Zhao et al., 2021). Mesoporous bioactive glasses (MBGs) with highly ordered nanoporous channels, large surface area, and high pore volume exhibited improved degradability and bioactivity compared with conventional bioglasses. Ternary composite scaffolds MGPC, including microfibrous mesoporous calcium silicate (mMCS), graphene oxide (GA), and PCL were 3D-printed and evaluated for compressive strength, *in vitro* degradability, and cell responses. Additionally, the *in vivo* osteogenesis and degradability of MGPC scaffolds were investigated through implantation in rabbit femur defects. The results showed that MGPC enhanced osteogenesis and had great potential for bone tissue engineering (Zhang et al., 2018).

Overall, this systematic review and meta-analysis revealed that 3D-printed composite scaffolds hold a promising and effective option in addressing bone defects of femur and tibia in animal models.

## 5 Limitations

3D-printed scaffolds have been regarded as a promising alternative for addressing bone repair in animal models. This study has several limitations including heterogeneities in study designs, lack of comparable groups (control vs. experimental groups), species variability, and short-term follow-up. In this systematic review and meta-analysis, there is variation in the animal models, for example, animal species, gender, age, and strain to study the impacts of composite scaffolds on bone regeneration at the defect site. Rabbits and rats were commonly utilized models without a distinct gender preference. However, there was considerable variability in the age of the animals, a crucial factor affecting the repair and regeneration of bone. Additionally, small animal models like rats and rabbits are commonly employed in this study, the inclusion of larger animal models such as sheep, goats, and pigs are recommended for future investigations. Overall, the outcomes are associated with bone tissue regeneration exhibit variability influenced by several key factors: the specific biomaterials employed, additional features such as porosity, inclusion of osteogenic factors, and the choice of animal model utilized.

The resemblance between clinical conditions in humans and these animal models is crucial for studying interactions with bone scaffolds. There is a lack of standardized protocols for scaffold fabrication and evaluation in animal models (femur and tibial defects) which may hinder the ability to draw definitive conclusions. Standardization issues can affect the reproducibility of results and limit the generalizability of findings due to variations in defect size, no. of defects, and evaluation time. There is another issue of publication bias as positive result are more likely to be published than studies with neutral or negative findings. This bias impacts the overall assessment of the effectiveness of 3D-bio-printed scaffolds. This systematic review also highlights a gap in the clinical translation of 3D-bio-printed scaffolds. Challenges and limitations in transitioning from animal models to human applications should be considered in the context of the review. Finally, human research must prioritize conducting high-quality clinical trials. These trials will yield the necessary evidence to comprehensively evaluate the effectiveness of 3D printing composite scaffolds in comparison to conventional grafting approaches.

## 6 Conclusion

In conclusion, this systematic review and meta-analysis of 3D-bio-printed scaffolds for BTE in animal models of femoral and tibial defects reveal promising advancements in the field with enhanced efficacy and safety. The fabricated 3D printed scaffolds exhibit a substantial potential for promoting bone regeneration, showcasing the versatility and adaptability of 3D-bio-printing technology in the context of BTE. However, it is crucial to acknowledge certain gaps in our current understanding and areas for future exploration. Further research is warranted to optimize the design and composition of 3D-bio-printed scaffolds, considering factors such as biomaterial selection, structural intricacies, and the incorporation of bioactive agents. Additionally, long-term studies assessing the stability, integration, and functional outcomes of these scaffolds in diverse animal models are essential. Future prospects in this field include a more comprehensive understanding of the immune response to 3D-bio-printed implants. Additionally, personalized approaches to scaffold design, tailored to meet individual patient-specific needs, are on the horizon. The ultimate goal is to effortlessly translate these advancements from animal models to practical clinical applications. Collaborative efforts among researchers, clinicians, and bioengineers will be instrumental in realizing the full potential of 3D-bio-printed scaffolds for BTE, ultimately contributing to improved treatments and outcomes for patients with bone defects. There is a need for well-designed multicentre randomized clinical trials to validate these findings and assess the cost-effectiveness of 3D printing. Such trials would contribute to a more comprehensive understanding and enhance the overall benefits of 3D printing in patient care.

## Data Availability

The raw data supporting the conclusions of this article will be made available by the authors, without undue reservation.
