# Supplementary material for: Unleashing innovation: 3D-printed biomaterials in bone tissue engineering for repairing femur and tibial defects in animal models – a systematic review and meta-analysis
Source: Front Bioeng Biotechnol. 2024 Sep 23;12:1385365. doi: 10.3389/fbioe.2024.1385365 (PMC11462855; doi:10.3389/fbioe.2024.1385365)
Supplement: Supplementary file 1 [file Table1.docx]

**Table 1:** Methodological characteristics of the studies included in the systematic reviews and characteristics of used scaffolds/template/biomaterials (n=37 studies)

| S.No. | Scaffolds/  biomaterials  /template | Scaffold analysis | Printing Technique | Cells (Cell Viability)/Growth factors | Pore Size/Pore width/Scaffold size/Membrane thickness  (µM) | Microporosity | Macroporosity | Total open porosity (%) | Compressive strength/ Tensile strength (MPa) | Elastic modulus/  Young modulus  (MPa) | Shrinkage/Bulk density/  Yield strength | References |
| --- | --- | --- | --- | --- | --- | --- | --- | --- | --- | --- | --- | --- |
| 1 | SiO2 and ZnO doped β-TCP | SEM  FESEM | Direct inkjet 3D printing | NG | 317 | 28.25% | 35.5% | - | - | - | - | [39] |
|  | β-TCP |  |  |  | - | 5.64% | - | - | - | - | - |  |
| 2 | Microwave-sintered 3D printed TCP scaffolds | SEM  FESEM  XRD | Direct 3D printing | Human Osteoblast Cell line | 400 | - | - | 42 | 10.95 ± 1.28 | - | 19-26% | [45] |
|  | Conventional sintered 3D printed TCP scaffold |  |  |  | 400 | - | - | 63 | 6.62 ± 0.67 | - | 11-18% |  |
|  | TCP scaffold |  |  |  | 500 | - | - | 27 | - | - | - |  |
| 3 | Bioceramic customized cage | XRD | 3D Printing | NG | 844 | 40% | - | 59.2 | 6.77 ± 1.22 | 6577 ± 1641 | - | [49] |
|  | TCP |  |  |  | 845 | - | - | - | 18.48 ± 3.40 | 1091 ± 153 | - |  |
| 4 | SrO- and MgO- in 3D printed  Microwave-sintered TCP scaffolds | FESEM  SEM  XRD | 3D printing | NG | 348±4.5 |  |  | 37.01±3.89 | 12.01 ± 1.56 | - | 52.84±2.76 | [26] |
|  | Conventional sintered SrO- and MgO- in 3D printed  TCP scaffolds |  |  |  | 361±9.1 |  |  | 41.63±2.09 | 9.38 ± 1.86 | - | 45.06±3.01 |  |
|  | Pure TCP scaffolds |  |  |  | 500 |  |  |  |  |  |  |  |
| 5 | Akermanite (Ca2MgSi2O7) scaffold | SEM | 3D printing | NG | 280x280 | - | - | 53 | 71.2±6.7 | 500 | - | [16] |
|  | β-TCP porous bioceramic |  |  |  | - | - | - | 56 | 10.5±3.0 | ⁓240 | - |  |
| 6 | Group I | SEM | Robocasting additive manufacturing technique | BMSCs (>95%) | 400 | - | - | - | - | - | - | [17] |
|  | Group II |  |  |  | 600 |  |  |  |  |  |  |  |
|  | Group III |  |  |  | 900 |  |  |  |  |  |  |  |
|  | Group IV (non-printed scaffold) |  |  |  | - |  |  |  |  |  |  |  |
|  | Group N (without scaffold) |  |  |  | - |  |  |  |  |  |  |  |
| 7 | PLA-HA composite scaffolds  (PLA 85% + HA 15%) + IVB  (Scaffold structure: 5-mm diameter, 6-mm height) | NG | Mini-deposition system (MDS)-3D printing | BMSCs | 500 | - | - | 60 | - | - | - | [23] |
| 8 | 30 MGPC (mMCS, GA, and PCL) | SEM  FE-SEM  XRD | 3D printing | MC3T3-E1 | 300 | - | - | 78.4 | 12.1 | - | - | [55] |
|  | 15 MGPC |  |  |  | 500 | - | - | 77.6 | 10.5 | - | - |  |
|  | GPC |  |  |  | 500 | - | - | 78.1 | 8.4 | - | - |  |
| 9 | Fe^+3^ and Si^+4^ Doped β-TCP | SEM  XRD | 3D Printing | NG | 300-310 | - | - |  | 19.8±2.4 | - | 16.76±3.4 | [43] |
|  | Fe^+3^ doped β-TCP |  |  |  | 310-330 | - | - |  | 17.9±1.3 | - | 17.89±0.98 |  |
|  | β-TCP |  |  |  | 340-350 | - | - |  | 4.9±0.7 | - | 10.00±2.1 |  |
| 10 | SiO2 and ZnO doped TCP | FESEM | 3D printing | NG |  |  |  |  | 10.21 ± 0.11 |  | 94.1 ± 1.6 | [44] |
|  | TCP |  |  |  |  |  |  |  | 5.48 ± 0.04 |  | 90.8 ± 0.8 |  |
| 11 | 3D Porous Bone Substitute Base  d on Calcium Phosphate | NG | 3D printing | NG | 300 | - | - | 55 | - | - | - | [48] |
| 12 | 3DPT (3D-printed PEEK with Ti) | XRD | 3D printing  (Extrusion method or FDM | MC3T3-E1 (>80%) | - | - | - | - | 84.1 | 2.42GPa | 78.7 | [70] |
|  | 3DP (3D-printed PEEK) |  |  |  |  |  |  |  |  |  |  |  |
|  | machined PEEK with Ti (MPT) |  |  |  |  |  |  |  |  |  |  |  |
|  | Machined PEEK (MP) |  |  |  |  |  |  |  |  |  |  |  |
| 13 | sodium alginate and CaCl2 (ratio-1:1) + addition of 10% (w/v) PEGDA, 5% (w/v) GelMA, and 0.05% (w/v) I-2959 | NG | Robotic in situ 3D bio-printing (extrusion-based) | MC3T3-E1 | - | - | - | - | Compression limit: 33.89%  Toughness: 71.35 kJ/m3 | 78.1 kPa | Stress limit: 60.46 kPa | [24] |
| 14 | AKT-H-3N | SEM  XRD | 3D printing | BMSCs | - | - | - | 72.98 |  |  |  | [18] |
|  | AKT-H-2N |  |  |  | - | - |  | 73.21 |  |  |  |  |
|  | AKT-H-1N |  |  |  |  |  |  | 70.59 |  |  |  |  |
|  | AKT-H |  |  |  |  |  |  | 72.83 |  |  |  |  |
|  | AKT |  |  |  |  |  |  | 57.65 |  |  |  |  |
| 15 | PLA and SDF-1 or BMP-7 immobil  ized in collagen type I. | - | 3D printing | NG | NG | - | - |  |  |  |  | [58] |
| 16 | PCL  Scaffold Combined with Co-Axially Electrospun  Vancomycin/  Ceftazidime/  Sheath-Core Nanofibers | SEM | 3D printing | BMP-2 | Average diameters: 111.68 ± 45.11 nm |  |  | 54.23 |  |  |  | [63] |
|  | PLGA/  vancomycin/  ceftazidime nanofibers |  |  |  | Average diameters: 1.58 ± 0.54 μm, |  |  | 73.68 |  |  |  |  |
|  | PLGA nanofibers |  |  |  | Average diameters: 1.04 ± 0.38µM |  |  | 61.12 |  |  |  |  |
| 17 | PLA scaffold with Biogel composed of gelatin and alginate | - | 3D printing | MSCs/rhBMP-2 | NG |  |  |  |  |  |  | [61] |
|  | PLA scaffold filled with Biogel (P-BG-M) |  |  | MSCs |  |  |  |  |  |  |  |  |
|  | PLA scaffold loaded on Biogel (P-BG-B2) |  |  | BMP-2 |  |  |  |  |  |  |  |  |
| 18 | PTMC+HA | SEM  XRD | 3D printing | MC3T3-E1, MSCs, K7M2, RAW264.7 /BMP, and zoledronic acid. | - |  |  | 70-73 |  |  | Residual masses:  53 ± 1% | [25] |
|  | PTMC |  |  | MC3T3-E1, MSCs, K7M2, RAW264.7 | - | - |  | 76 |  |  | Residual masses:  54 ± 1% |  |
| 19 | Gene-activated implants based on octacalcium phosphate (OCP) and plasmid DNA encoding *VEGFA*. | SEM  XRD  FTIS | 3D printing | NG | - | - | - | - | 1.5-4.5 MPa | - | - | [50] |
| 20 | PCL scaffolds fibrin-based hydrogel, gelatin methacrylamide, fibrin and alginate | SEM | 3D printing | hBMSCs (⁓80%) HUVECs | NG | - | - |  |  |  |  | [71] |
| 21 | PLA 100M^+β-TCP^ | SEM | 3D printing | MSCs | - | Crystallinity: 0.05% | Weight: 301 mg ± 4.5 SD |  |  | 319.63±2.77 |  | [19] |
|  | PLA Pure Polymer (100M) |  |  |  | 750 mm | Crystallinity: 0.2% | Weight:  240.6 mg ±1.5 SD |  |  | 304.56±44.69 |  |  |
|  | 7415^+ β-TCP^ |  |  |  |  | Crystallinity: 0.32% | Weight:  28 mg ± 0.8 SD |  |  | 94.56 ± 7.9 |  |  |
|  | 7415 |  |  |  |  | Crystallinity: 0.09% | Weight: 216 mg ± 2 SD |  |  | 62.56 ± 12.95 |  |  |
| 22 | CpTi-P | FESEM | 3D printing (DED technique) | hFOB |  |  |  |  | 1220 | 127±11 GPa | 603±9 | [72] |
|  | 10Ta-P |  |  |  |  |  |  |  | 1310 | 79±5 GPa | 803±70 |  |
|  | 25Ta-P |  |  |  |  |  |  |  | 1180 | 64±6 GPa | 686±13 |  |
|  | 100Ta-P |  |  |  |  |  |  |  | 560 | 98±4 GPa | 277±2 |  |
| 23 | HA/PLGA copolymer | SEM | 3D printing |  |  |  |  |  |  |  |  | [62] |
| 24 | BGS  (SiO2: CaO: P2O5 = 35:50:15) | SEM | 3D printing | NG | Microspheres size: ⁓ 350 ± 38 nm |  |  |  |  |  |  | [69] |
| 25 | GelMA Scaffolds (5% w/v) | SEM | 3D printing | BMSCs (68%)/BMP-2 | 180–240 |  |  |  | 33.63 ± 7.57 kPa |  | 24.07 ±  6.18 kPa | [20] |
|  | GelMA Scaffolds (10% w/v) |  |  |  | 90–130 |  |  |  | 66.16 ± 10.13 kPa |  | 40.47 ± 6.36 kPa |  |
|  | GelMA Scaffolds (15% w/v) |  |  |  | 40–60 |  |  |  | 96.65 ± 14.15 kPa |  | 60.97 ± 6.07 kPa |  |
| 26 | graphene-containing (1, 3, 5, 10 wt%), porous and oriented poly-ε-caprolactone-based scaffolds |  | 3D printing robocasting method |  |  |  |  |  |  |  |  | [65] |
| 27 | Gelatin/PCL membrane as a GBR construct | SEM | 3D printing | Fibroblast and Saos-2 cells (>80%) | Membrane thickness: 500µM |  |  |  |  |  | 11.8 ± 2.7 Mpa | [73] |
|  | PCL |  |  |  | Membrane thickness: 400µM |  |  |  |  |  | 12.3 ± 1.6 Mpa |  |
| 28 | hydroxyapatite (HA) scaffolds |  | 3D printing |  |  |  |  |  |  |  |  | [51] |
| 29 | IONPs | SEM | 3D printing | PCSCs (>95%) | Average size: 34.9 nm | - | **-** | - | - | - | - | [74] |
|  | Iron oxide core |  |  |  | Average size: 7.14 ± 0.68 nm. | - | **-** | - | - | - | - |  |
| 30 | icariin-loaded Ti6Al4V reconstruction rod | SEM  Confocal microscopy | 3D printing | MC3T3-E1 | - | - | - | - | - | - | - | [21] |
| 31 | Fibrin-based bioinks and printed PCL frameworks: fibrinogen, type A gelatin, HA, and glycerol-based bio-ink | SEM | 3D printing (FDM) | hMSCs (>94%) | - | - | - | - | - | - | - | [75] |
| 32 | PLGA (ratios of LA: GA-65:35 and 75:25) and blended with graphene nanoparticles | SEM | 3D printing | human ADMSCs transduced with RFP | - | - | - | - | - | - | - | [67] |
| 33 | RP scaffold | SEM | 3D printing | Embryonic MSCs | Scaffold size: 6 mm in height and 5 mm in diameter | - | - | 50 | 6.6 ± 0.8 | - | - | [76] |
|  | SSM scaffold |  |  |  | 200–300 | - | - | 86.9 | 0.46 ± 0.2 | - | - |  |
| 34 | HA 3D-printed scaffolds with Gyroid-TPMS | SEM | 3D printing | - | - | - | - | 65 | 4.29 ±0.35 | - | - | [76] |
| 35 | PDA-β-TCP/PCL composite scaffolds | SEM | 3D printing | MSCs | - | - | - | - | - | - | - | [22] |
| 36 | HA/β-TCP/SF | SEM  XRD  FTIR spectroscopy | 3D printing | MC3T3-E1 | - | - | - | - | - | - | - | [47] |
| 37 | GO-PCL scaffolds | SEM  XRD | 3D printing | PBMCs (79.36 ± 5.29) | 335±20.01 | - | - | 50 ± 3.3 | - | - | - | [66] |

MBG: mesoporous bioglass, MCS: magnesium calcium silicate, GA: gliadin, PCL: polycaprolactone, FTIS: Fourier transform infrared spectroscopy, BGS: Bioactive glass, scaffold, PCL: poly-ε-caprolactone, RFP: red fluorescent protein, TPMS: Triply periodic minimal surface, GO: graphene oxide, PCSCs: precartilaginous stem cells, IONP: Iron oxide nanoparticles, PLGA: poly(lactic-glycolic acid)
