# Supplementary material for: Unleashing innovation: 3D-printed biomaterials in bone tissue engineering for repairing femur and tibial defects in animal models – a systematic review and meta-analysis
Source: Front Bioeng Biotechnol. 2024 Sep 23;12:1385365. doi: 10.3389/fbioe.2024.1385365 (PMC11462855; doi:10.3389/fbioe.2024.1385365)
Supplement: Supplementary file 2 [file Presentation1.pptx]

## Slide 1
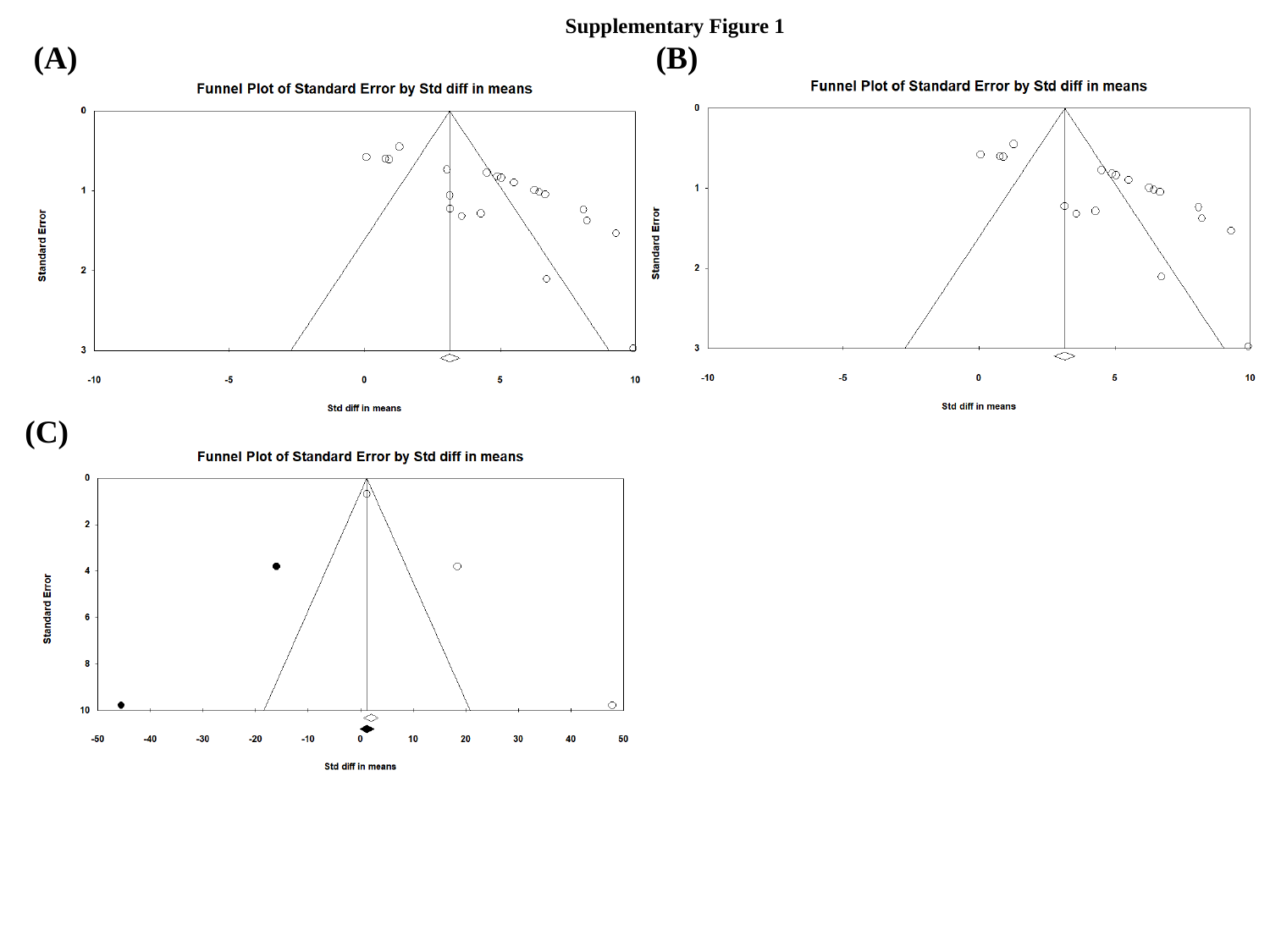

Supplementary Figure 1
(A) (B)
(C)

## Slide 2
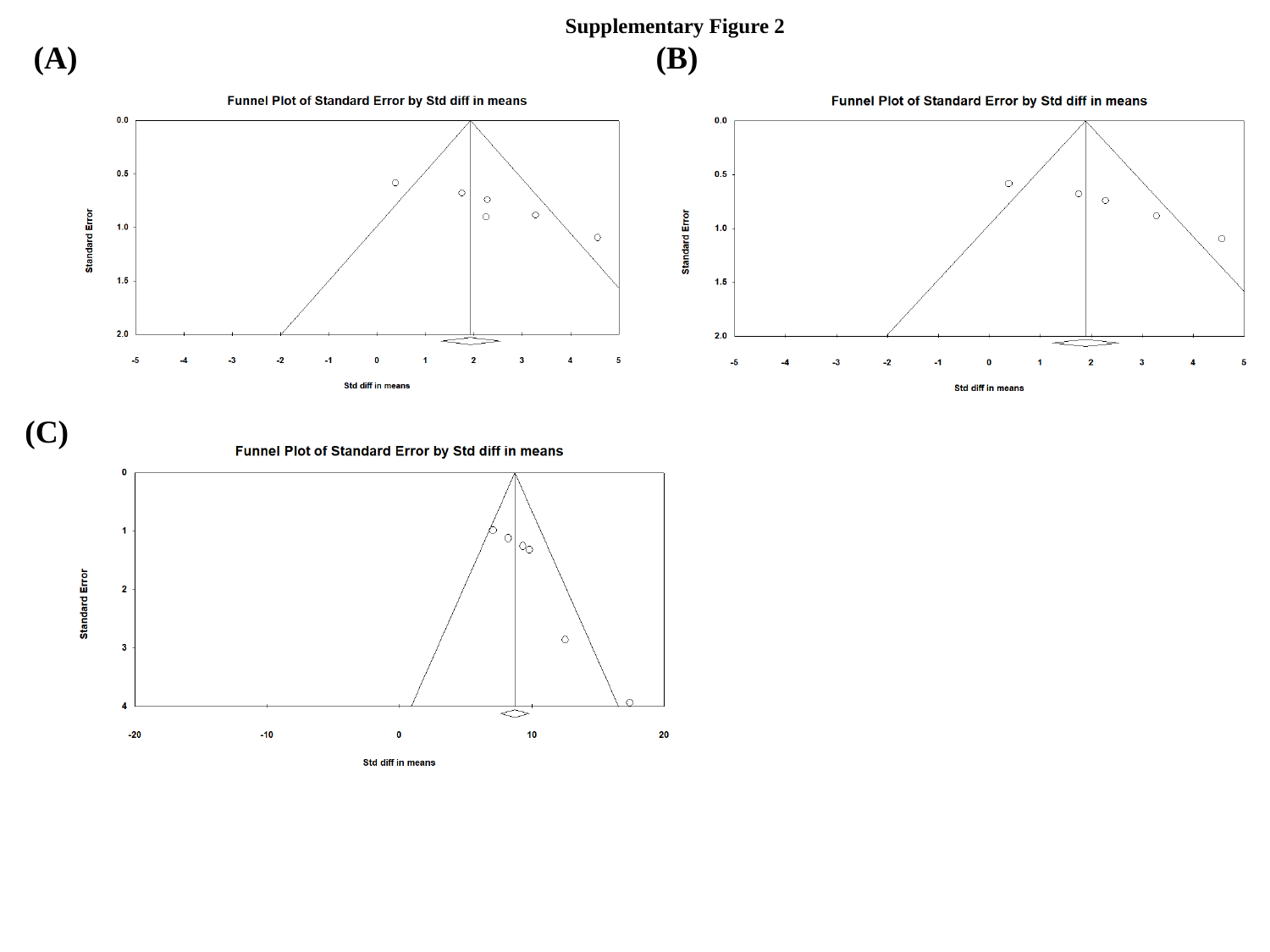

Supplementary Figure 2
(A) (B)
(C)

## Slide 3
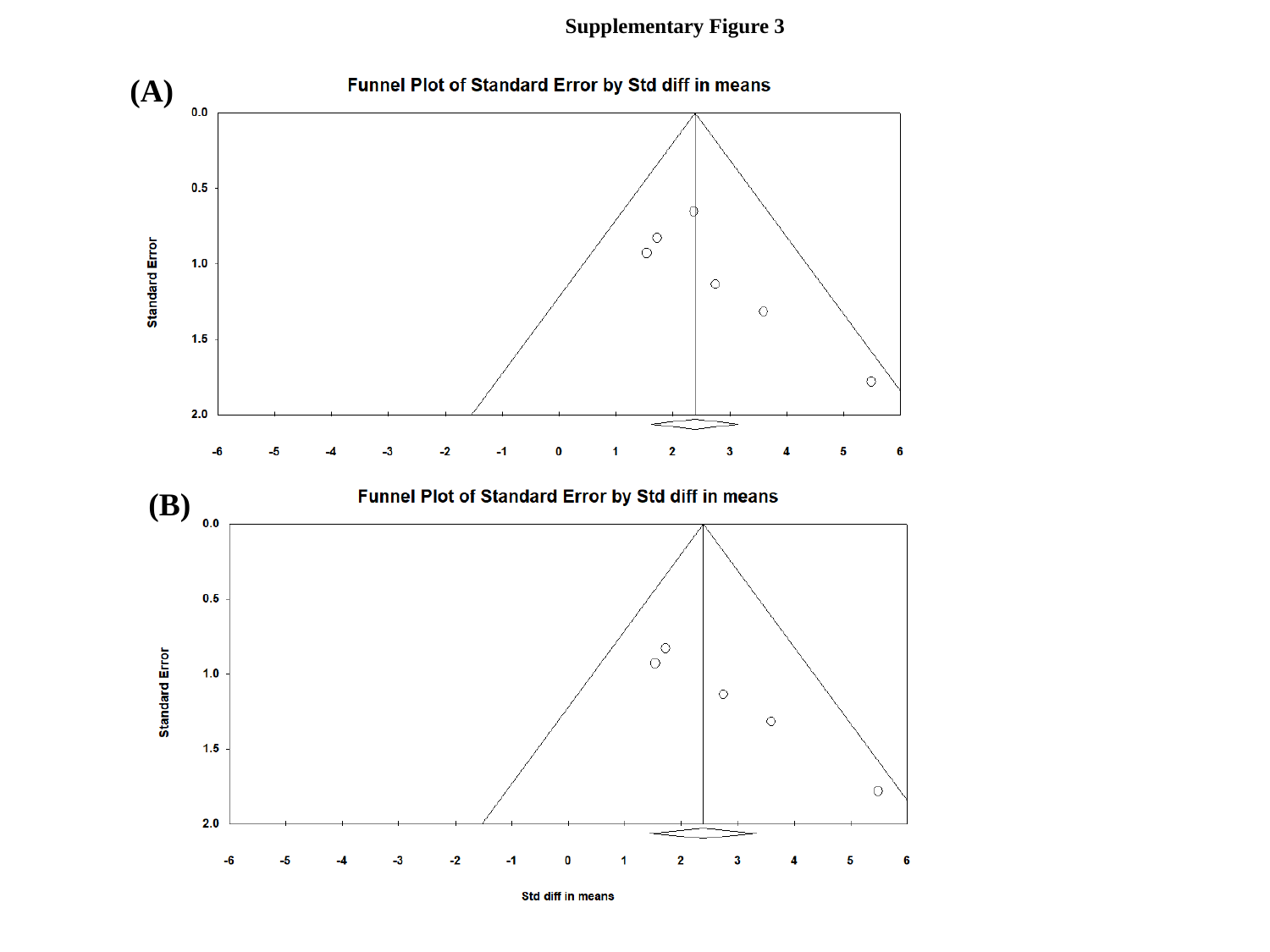

Supplementary Figure 3
(A)
(B)

## Slide 4
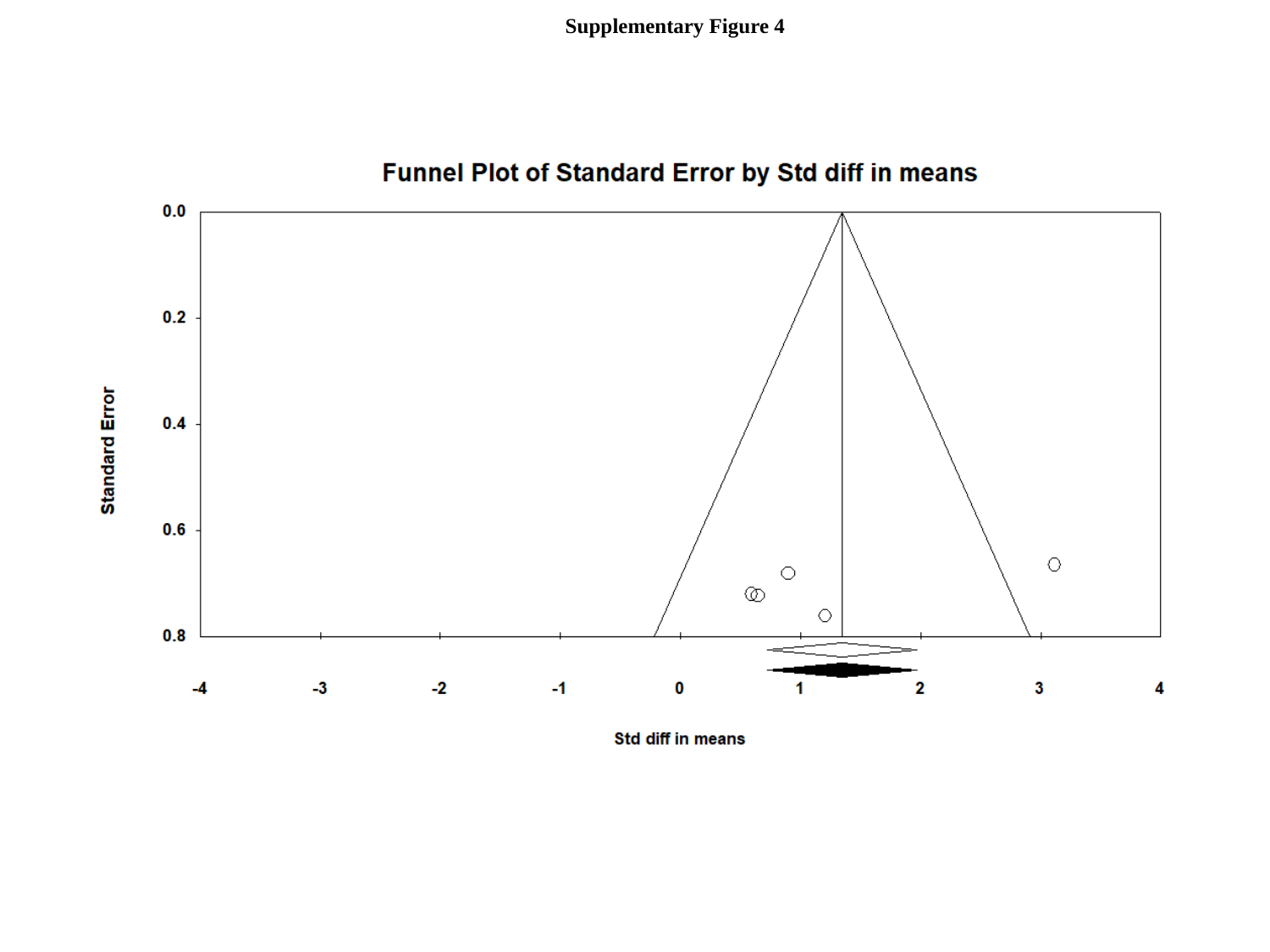

Supplementary Figure 4
